# Exploring the hidden riches: Recent remarkable faunistic records and range extensions in the bee fauna of Italy (Hymenoptera, Apoidea, Anthophila)

**DOI:** 10.3897/BDJ.12.e116014

**Published:** 2024-02-16

**Authors:** Maurizio Cornalba, Marino Quaranta, Marco Selis, Simone Flaminio, Sirio Gamba, Maurizio Mei, Marco Bonifacino, Andree Cappellari, Roberto Catania, Pietro Niolu, Stefano Tempesti, Paolo Biella

**Affiliations:** 1 Department of Mathematics, University of Pavia, Pavia, Italy Department of Mathematics, University of Pavia Pavia Italy; 2 Centro di Ricerca Agricoltura e Ambiente, (CREA) Consiglio per la Ricerca in Agricoltura e l’analisi dell’Economia Agraria, Bologna, Italy Centro di Ricerca Agricoltura e Ambiente, (CREA) Consiglio per la Ricerca in Agricoltura e l’analisi dell’Economia Agraria Bologna Italy; 3 Via dei Tarquini, Viterbo, Italy Via dei Tarquini Viterbo Italy; 4 Laboratory of Zoology, Research Institute for Biosciences, University of Mons, Monsa, Belgium Laboratory of Zoology, Research Institute for Biosciences, University of Mons Monsa Belgium; 5 Strada Sanferian, San Biagio della Cima (Imperia), Italy Strada Sanferian San Biagio della Cima (Imperia) Italy; 6 Department of Biology and Biotechnology “Charles Darwin”, Sapienza University of Rome, Rome, Italy Department of Biology and Biotechnology “Charles Darwin”, Sapienza University of Rome Rome Italy; 7 Department of Biology, University of Florence, Sesto Fiorentino, Italy Department of Biology, University of Florence Sesto Fiorentino Italy; 8 Department of Agronomy, Food, Natural resources, Animals and Environment (DAFNAE), University of Padova, Padova, Italy Department of Agronomy, Food, Natural resources, Animals and Environment (DAFNAE), University of Padova Padova Italy; 9 Department of Agriculture, Food and Environment (Di3A), sec. Applied Entomology, Catania, Italy Department of Agriculture, Food and Environment (Di3A), sec. Applied Entomology Catania Italy; 10 Via Sassari, Alghero, Italy Via Sassari Alghero Italy; 11 Via Vincenzo Bellini, Santa Sofia (Forlì-Cesena), Italy Via Vincenzo Bellini Santa Sofia (Forlì-Cesena) Italy; 12 Department of Biotechnology and Biosciences, University of Milano-Bicocca, Milano, Italy Department of Biotechnology and Biosciences, University of Milano-Bicocca Milano Italy

**Keywords:** DNA barcoding, species distribution, rare species of bees, sampling in neglected areas, lack of taxonomists, biodiversity of Italian Peninsula, Hymenoptera

## Abstract

**Background:**

The area sourrounding the Mediterranean basin is recognised as a major biodiversity hotspot for bees, and Italy is amongst the European countries with the highest bee species richness. Detailed knowledge of bee distribution is crucial for understanding bee biology and designing tailored conservation strategies, but is still insufficient in southern European countries, especially in Italy.

**New information:**

We report recent finds of 48 bee species that yield significant novelties for the Italian bee fauna. Eight species, namely *Andrenaconfinis* Stöckhert, *Anthidiellumbreviusculum* Pérez, *Coelioxysalatus* Foerster, *Lasioglossumalgericolellum* Strand, *Megachilelapponica* Thomson, *Megachileopacifrons* Pérez, *Megachilesemicircularis* auct. nec Zanden and *Trachusaintegra* Eversmann are reported as new for Italy. In addition, *Andrenabinominata* Smith, *Andrenacompta* Lepeletier, *Colletesacutus* Pérez, *Lasioglossumstrictifrons* Vachal, *Rhodanthidiumsiculum* Spinola and *Rhodanthidiumsticticum* Fabricius are newly recorded from mainland Italy, *Osmiaheteracantha* Pérez from Sardegna and *Nomadaflavopicta* Kirby from Sicilia. We also report significant range extensions for other bee species and recent records of species that had long gone unrecorded in Italy. The combination of morphology and DNA barcoding provided reliable identifications even for the most challenging specimens. As several of our records come from areas neglected by bee experts in the past, this study stands out as a key indicator of a bee faunistic richness still awaiting discovery and hopefully it will stimulate the interest of taxonomists and stakeholders in pursuing bee research in Italy in the near future.

## Introduction

The Mediterranean Basin is one of the major hotspots for bee diversity (Michener 2007, Nieto et al. 2014, Orr et al. 2021). Italy, in particular, has one of the richest bee faunas in the world by land area, with well over 1000 confirmed species (Pagliano 1995, Comba 2019, Ghisbain et al. 2023, Reverté et al. 2023). However, this fauna remains insufficiently known in comparison with the ones of nearby Central European and Mediterranean regions, which have received considerably more attention, especially in recent years. The coverage of the Italian territory is markedly uneven, with large areas only occasionally investigated or still virtually unexplored. Moreover, published data are sometimes of poor quality and unreliable and recent ones are often still unpublished and difficult to access. This makes it hard to design and implement effective conservation actions. For instance, these factors significantly limited the evaluation of the conservation status of several potentially threatened Italian bee species (Quaranta et al. 2018).

It was primarily the awareness of the problems sketched above that led to the creation in 2022 of an informal network of Italian bee enthusiasts. The primary goal of the network is to contribute to a better knowledge of the Italian bee fauna, through the exchange of data, images and specimens and the collective discussion of critical determination cases. The present paper details the main results obtained by the network during its first year of activity. We report records of 368 specimens belonging to 48 bee species. Eight of the species are new for the bee fauna of Italy and, in addition to these, six are new for mainland Italy, one for Sardegna and one for Sicilia; our data form the basis for the inclusion of these species in the Italian, Sardinian and Sicilian checklists (Reverté et al. 2023). We also report biogeographically significant extensions of the Italian ranges of thirty species. Finally, we document the continuing occurrence in Italy of a few species which had gone unrecorded in the country for a long time. A central aspect of our work is that it combines morphological identifications with the use of DNA barcoding targeting the mitochondrial COI region, which played a significant role in our study in at least two ways. On the one hand, in several cases, barcoding provided additional confirmation of morphological identifications. On the other hand, for some species, the DNA sequences obtained here constitute the first COI barcodes ever produced.

## Materials and methods

The collections studied in the present paper are the personal ones of the authors, supplemented by a very limited number of specimens collected and/or determined by the authors and deposited in other collections. In addition, four records, two of which crucial, were generously communicated by Christophe Praz.

The records selected for publication are primarily those of the species that appear to be new for Italy or for one of its administrative regions. To rule out the existence of previous records, in addition to carefully and critically searching the literature, we critically explored online resources like the Italian catalogue (Comba 2019), Discover Life (Ascher and Pickering 2023), Westpalbees (Kuhlmann et al. 2012), Palaearctic Osmiine Bees (Müller 2022), GBIF (www.gbif.org), iNaturalist (www.inaturalist.org) and Naturgucker (www.naturgucker.info). We provide maps for the species for which our data indicate very significant range extensions. In the species accounts, we use WGS84 decimal degree coordinates and the date format yyyy-mm-dd.

The maps for the Italian records of selected species were drawn with QGIS 3.22.16 (https://www.qgis.org/en/site/index.html). Regional and country boundaries were obtained from Istat (https://istat.it/it/archivio/222527) and Eurostat (https://ec.europa.eu/eurostat/web/main/data, ©EuroGeographics for the administrative boundaries). The background Digital Terrain Model was obtained from https://data.europa.eu/data/datasets/m_amte-299fn3-eba41113-4141-4d46-9cdf-b0848deec44d?locale=it.

Photographs were taken with four different kinds of equipment. The authors used a Nikon D500 camera, mounted on a Nikon bellows with an inverted, f/2.8-50mm, Rodenstock Rodagon lens and NiSi NM180 macro focusing rail; or a Canon Eos 60D camera with a Sigma 150 mm f/2.8 EX DG OS HSM Macro lens; or a Canon EOS 1300D camera equipped with an inverted Canon EF-S 18-55 mm lens and extension tubes; or a Zeiss Axio Zoom V16 microscope with a Plan Z 1.0/0 FWD 60 mm lens integrated with Axiocam 807. Stacking of pictures was performed with Helicon Focus (HeliconSoft) or CombineZP software.

### Abbreviations of entomological collections

CEUSS = Entomological Collection of the University of Sassari, Sassari, Italy

CPC = private collection of Christophe Praz, Neuchâtel, Switzerland

MBC = private collection of Marco Bonifacino, Vado Ligure

MCC = private collection of Maurizio Cornalba, Pavia, Italy

MEC = private collection of Maurizio Mei, Rome, Italy

MIB:ZPL = Collection of the ZooPlantLab of the University of Milano-Bicocca, Milan, Italy

MKC = private collection of Michael Kuhlmann, Kiel, Germany

MSC = private collection of Marco Selis, Viterbo, Italy

MZUR = Museum of Zoology of the Sapienza University of Rome, Rome, Italy

RCC = private collection of Roberto Catania, Catania, Italy

SFC = private collection of Simone Flaminio, Bologna, Italy

SGC = private collection of Sirio Gamba, San Biagio della Cima, Italy

### Taxonomic framework

We follow the taxonomy adopted in the new checklist of the wild bees of Europe (Ghisbain et al. 2023). In particular, we view *Seladonia* Robertson (including *Pachyceble* Moure) as a valid genus distinct from *Halictus* Latreille, and we include the West Palaearctic lineages of *Tetraloniella* Ashmead in the genus *Tetralonia* Spinola. Most morphological identifications were carried out by the authors using standard keys and the relevant specialized literature (e.g. Friese (1897), Noskiewicz (1936), Benoist (1940), Ebmer (1971), Ebmer (1975), Tkalců (1978), Tkalců (1979), Dathe (1980), Warncke (1980), Warncke (1983), Ebmer (1986), Schmid-Egger and Scheuchl (1997), Herrmann and Doczkal (1999), Dathe (2000), Amiet et al. (2001), Gusenleitner and Schwarz (2002), Amiet et al. (2004), Michez and Patiny (2005), Schmid-Egger (2005), Scheuchl (2006), Amiet et al. (2007), Chorein (2007), Pauly (2009), Amiet et al. (2010), Dathe et al. (2016), Smit (2018), Bogusch and Hadrava (2018), Kasparek (2019), Praz et al. (2019), Aubert (2020), Kasparek (2020), Litman et al. (2021), McLaughlin et al. (2022)) and, in many cases, by direct comparison with safely identified reference specimens. All determinations were critically discussed within the authors’ network. Some critical specimens were identified by leading specialists, who also validated several of the identifications made by the authors.

### DNA barcoding

In several cases, particularly for difficult-to-determine species, the morphological identifications were confirmed by DNA barcoding. To this end, total DNA was extracted from a leg of one or more specimens using a DNeasy Blood & Tissue Kit (Qiagen, Milan, Italy). The COI standard barcode region was targeted to obtain DNA barcodes (i.e. 658 bp at the 5’ end of the mitochondrial COI). To do so, the standard barcoding primers LCO1490/HCO2198 were used. The taxonomic identity of the processed bees was tested by comparing the COI sequences thus obtained with the reference DNA barcode bank stored in the Barcode of Life Database (BOLD System) using the Identification Engine tool (IDS) (http://www.boldsystems.org/index.php/IDS_IdentificationRequest; Species Level Barcode Records database). To obtain reliable identifications, the species lists, the matching rates and the neighbour-joining tree returned by IDS were scrutinised for each submitted sequence, in particular by verifying the presence of multiple concordant identification outputs of very high similarity scores and/or sequences forming well defined clades with a high majority of concordant sequences names in the neighbour-joining tree. All specimens subjected to DNA barcoding are listed in Suppl. material 1. Sequences are deposited in BOLD Systems (project "RApiTI - Italian Rare Bees" [ZPLRP]).

## Checklists

### Annotated list of significant records

#### Colletes acutus

Pérez, 1903

AA265FBB-D668-5FC8-B37F-C8B3530BDF9A

##### Materials

- **Type status:** Other material. **Occurrence:** recordedBy: Maurizio Bollino; sex: 2 females; occurrenceID: 710E7EAF-A075-5B97-83EB-6D202E24AAE3; **Location:** countryCode: IT; stateProvince: Puglia; county: Lecce; municipality: Vernole; locality: Termetito; verbatimElevation: 5 m; verbatimLatitude: 40.3391; verbatimLongitude: 18.365; **Identification:** identifiedBy: Marco Selis, vid. M. Kuhlmann; **Event:** eventDate: 2021-05-16/17; **Record Level:** collectionCode: MSC, MKC

##### Notes

**Figure 1.**
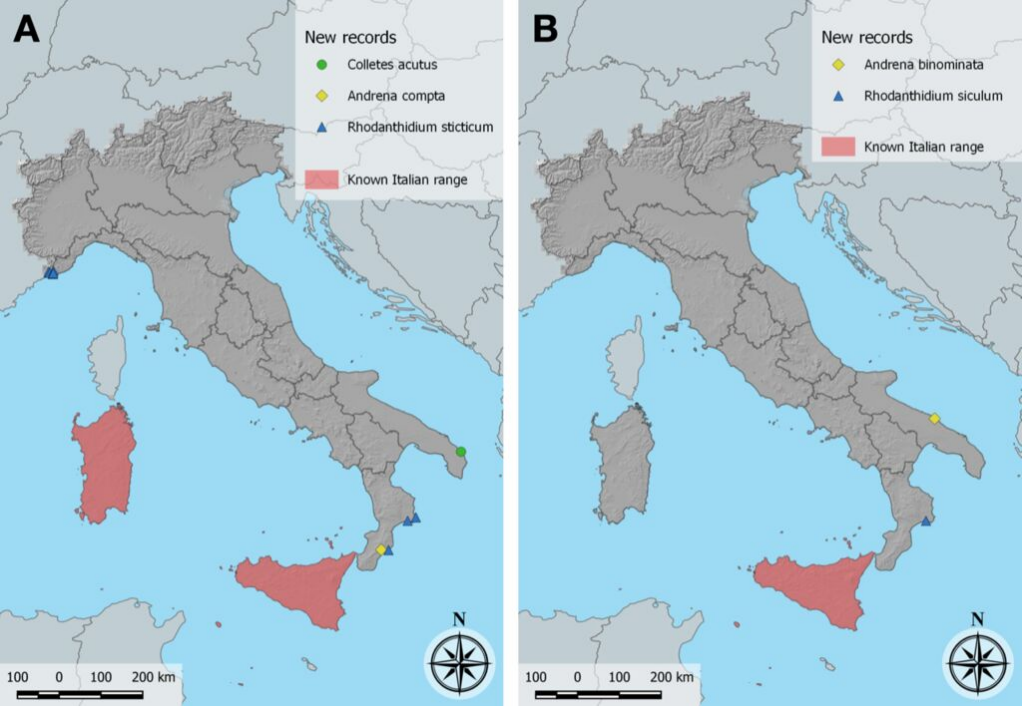
Known Italian range and new records of (A) *Colletesacutus* Pérez, 1903, *Andrenacompta* Lepeletier, 1841, *Rhodanthidiumsticticum* (Fabricius, 1787) and (B) *Andrenabinominata* Smith, 1853, *Rhodanthidiumsiculum* (Spinola, 1838).

Prior to our find, *C.acutus* was known in Italy only from Sardegna and Sicilia (Comba 2019 and references therein). The Puglia records are by far the easternmost known for the species, whose range they significantly extend (Fig. 1A).

#### Hylaeus glacialis

Morawitz, 1872

697E91F9-FAAE-5071-865E-BB4280E4A3F8

##### Materials

- **Type status:** Other material. **Occurrence:** recordedBy: Marco Selis; sex: 1 male; occurrenceID: 3E72290B-EBC9-5374-800E-E3318AD62F1C; **Location:** countryCode: IT; stateProvince: Lazio; county: Rieti; municipality: Lonessa; locality: Sella di Leonessa; verbatimElevation: 1700-1870 m; verbatimLatitude: 42.48; verbatimLongitude: 13.0075; **Identification:** identifiedBy: Marco Selis, vid. R. Le Divelec; **Event:** eventDate: 2021-07-11; **Record Level:** collectionCode: MSC

##### Notes

**Figure 2.**
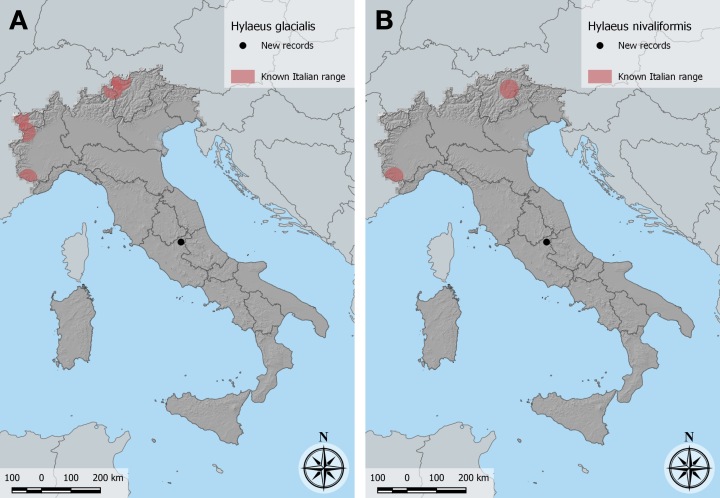
Known Italian range and new records of *Hylaeusglacialis* Morawitz, 1872 (A) and *Hylaeusnivaliformis* Dathe, 1977 (B).

This is the first record of *H.glacialis* for the Apennines. Previously known in Italy only from the Alps of Piemonte, Valle d’Aosta and Trentino-Alto Adige/Südtirol (Ebmer 2003, Schmid-Egger 2011, Praz et al. 2022; Fig. 2A).

#### Hylaeus nigrifacies

Bramson, 1879

96123092-D923-5968-96A9-3DE969ADBECA

##### Materials

- **Type status:** Other material. **Occurrence:** recordedBy: Simone Flaminio; sex: 2 females; occurrenceID: B0B8D67D-0E27-5CA9-A123-07449D8CA167; **Location:** countryCode: IT; stateProvince: Emilia-Romagna; county: Bologna; municipality: Bologna; verbatimLatitude: 44.4506; verbatimLongitude: 11.3675; **Identification:** identifiedBy: Romain Le Divelec; **Event:** eventDate: 2020-07-01; **Record Level:** collectionCode: SFC
- **Type status:** Other material. **Occurrence:** recordedBy: Marco Selis; sex: 1 female; occurrenceID: 80BE3B6E-E1AE-5CC3-B9D6-A3BD4F9E4E4D; **Location:** countryCode: IT; stateProvince: Lazio; county: Viterbo; municipality: Bomarzo; locality: fiume Tevere; verbatimElevation: 50 m; verbatimLatitude: 42.5131; verbatimLongitude: 12.2744; **Identification:** identifiedBy: Marco Selis, vid. R. Le Divelec; **Event:** eventDate: 2021-08-30; **Record Level:** collectionCode: MSC
- **Type status:** Other material. **Occurrence:** recordedBy: Marco Selis; sex: 4 males, 7 females; occurrenceID: FC3B343B-390F-58CB-87D1-FEB5DBCE5806; **Location:** countryCode: IT; stateProvince: Lazio; county: Viterbo; municipality: Bomarzo; locality: fiume Tevere; verbatimElevation: 50 m; verbatimLatitude: 42.5131; verbatimLongitude: 12.2744; **Identification:** identifiedBy: Marco Selis, vid. R. Le Divelec; **Event:** eventDate: 2022-07-2/10; **Record Level:** collectionCode: MSC

##### Notes

According to Le Divelec (2021), *Hylaeusnigrifacies* is the correct name for the species that has generally been known as *Hylaeusmoricei* (Friese, 1898). The only published record of *H.nigrifacies* from Italy is that of a specimen from Naples, one of the paratypes of *Prosopisnigrifaciesrhenana* Warncke, 1986 (Warncke 1986).

#### Hylaeus nivaliformis

Dathe, 1977

01501A23-DE94-5C8C-AC6E-B470913A04EE

##### Materials

- **Type status:** Other material. **Occurrence:** recordedBy: Marco Selis; sex: 1 male; occurrenceID: 811402E6-2B00-5550-89AC-53ED8F4D2E1B; **Location:** countryCode: IT; stateProvince: Lazio; county: Rieti; municipality: Lonessa; locality: Sella di Leonessa; verbatimElevation: 1700-1870 m; verbatimLatitude: 42.48; verbatimLongitude: 13.0075; **Identification:** identifiedBy: Marco Selis, vid. R. Le Divelec; **Event:** eventDate: 2021-07-11; **Record Level:** collectionCode: MSC
- **Type status:** Other material. **Occurrence:** recordedBy: Marco Selis; sex: 1 female; occurrenceID: B1098737-51AD-5952-ADC8-A033300B641E; **Location:** countryCode: IT; stateProvince: Lazio; county: Rieti; municipality: Lonessa; locality: Sella di Leonessa; verbatimElevation: 1700-1870 m; verbatimLatitude: 42.48; verbatimLongitude: 13.0075; **Identification:** identifiedBy: Marco Selis, vid. R. Le Divelec; **Event:** eventDate: 2021-07-28; **Record Level:** collectionCode: MSC
- **Type status:** Other material. **Occurrence:** recordedBy: Marco Selis; sex: 4 males, 1 female; occurrenceID: 295910CE-2B90-5FAD-918D-F1166EF97C5B; **Location:** countryCode: IT; stateProvince: Lazio; county: Rieti; municipality: Lonessa; locality: Sella di Leonessa; verbatimElevation: 1700-1870 m; verbatimLatitude: 42.48; verbatimLongitude: 13.0075; **Identification:** identifiedBy: Marco Selis, vid. R. Le Divelec; **Event:** eventDate: 2022-06-19; **Record Level:** collectionCode: MSC

##### Notes

Previously known in Italy only from the Eastern Alps in Südtirol and from the Maritime Alps in Piemonte (Dathe 2000). Our records of *H.nivaliformis* are the first for the Apennines (Fig. 2B).

#### Andrena alutacea

Stoeckhert, 1942

C07D429E-48CB-5045-BC33-B5C746785115

##### Materials

- **Type status:** Other material. **Occurrence:** recordedBy: Maurizio Cornalba; sex: 2 females; occurrenceID: 89534A0C-8297-5A7C-BD49-23D38C3B9AFD; **Location:** countryCode: IT; stateProvince: Lombardia; county: Bergamo; municipality: Piazzatorre; verbatimElevation: 829 m; verbatimLatitude: 45.9883; verbatimLongitude: 9.6795; **Identification:** identifiedBy: Maurizio Cornalba; **Event:** eventDate: 2022-07-14; **Record Level:** collectionCode: MCC
- **Type status:** Other material. **Occurrence:** recordedBy: Maurizio Cornalba; sex: 1 female; occurrenceID: 4746F05A-1737-5C4B-A4BE-B068576EFB41; **Location:** countryCode: IT; stateProvince: Lombardia; county: Bergamo; municipality: Piazzatorre; verbatimElevation: 840 m; verbatimLatitude: 45.9886; verbatimLongitude: 9.6803; **Identification:** identifiedBy: Maurizio Cornalba; **Event:** eventDate: 2022-07-14; **Record Level:** collectionCode: MCC
- **Type status:** Other material. **Occurrence:** recordedBy: Maurizio Cornalba; sex: 1 female; occurrenceID: 0FECB28F-3172-5CB4-8542-EF5E5E2715B8; **Location:** countryCode: IT; stateProvince: Lombardia; county: Bergamo; municipality: Piazzatorre; verbatimElevation: 840 m; verbatimLatitude: 45.9886; verbatimLongitude: 9.6803; **Identification:** identifiedBy: Maurizio Cornalba; **Event:** eventDate: 2022-07-16; **Record Level:** collectionCode: MCC
- **Type status:** Other material. **Occurrence:** recordedBy: Maurizio Cornalba; sex: 1 female; occurrenceID: 8B2F44DC-0BC1-5BC8-AA83-C54D79D848C0; **Location:** countryCode: IT; stateProvince: Lombardia; county: Bergamo; municipality: Piazzatorre; verbatimElevation: 841 m; verbatimLatitude: 45.9878; verbatimLongitude: 9.6813; **Identification:** identifiedBy: Maurizio Cornalba; **Event:** eventDate: 2022-07-31; **Record Level:** collectionCode: MCC

##### Notes

One specimen confirmed by DNA barcoding: the match with reference sequences is between 100% and 99.84% (mean: 99.96%). All specimens were collecting pollen on *Pimpinellamajor* in a hay meadow. Previously recorded in Italy in Trentino-Alto Adige (Schmid-Egger 2005) and in the Maritime Alps of Piemonte (Schmid-Egger 2011). The occurrence of the species in these two regions and, more specifically, in the Ligurian Alps, is mentioned also by Stoeckhert (1942). *A.alutacea* has recently been found to occur, or have occurred, also on Mount Etna in Sicilia (Wood et al. 2023).

#### Andrena amieti

Praz, Müller, Genoud, 2019

A610F762-E7D7-5DF6-B8E2-5506EEFD4731

##### Materials

- **Type status:** Other material. **Occurrence:** recordedBy: Marco Selis; sex: 1 female; occurrenceID: C841AD1C-06CB-55D4-BFB4-DFAE07E3BE6C; **Location:** countryCode: IT; stateProvince: Lazio; county: Rieti; municipality: Lonessa; locality: Sella di Leonessa; verbatimElevation: 1700-1870 m; verbatimLatitude: 42.48; verbatimLongitude: 13.0075; **Identification:** identifiedBy: Marco Selis, vid. T.J. Wood; **Event:** eventDate: 2021-07-28; **Record Level:** collectionCode: MSC
- **Type status:** Other material. **Occurrence:** recordedBy: Marco Selis; sex: 1 male, 1 female; occurrenceID: 3A847C05-22A0-51A3-8D4B-71EF918B8D19; **Location:** countryCode: IT; stateProvince: Lazio; county: Rieti; municipality: Lonessa; locality: Sella di Leonessa; verbatimElevation: 1700-1870 m; verbatimLatitude: 42.48; verbatimLongitude: 13.0075; **Identification:** identifiedBy: Marco Selis, vid. T.J. Wood; **Event:** eventDate: 2022-06-19; **Record Level:** collectionCode: MSC

##### Notes

In Italy, *Andrenaamieti* had previously been recorded throughout the Alps and on Monte Pollino in Calabria (Praz et al. 2019). Our find shows that it also occurs in the Central Apennines, as was to be expected. It is shown by Praz et al. (2019) that two divergent mitochondrial lineages are found in *A.amieti*. One lineage, called Group 1, is reported from Calabria and, sparingly, from the Alps. The other lineage occurs widely in the Alps. DNA barcoding of one of our specimens confirmed the morphological identification and showed that the specimen belongs to Group 1. The match with the two sequences from Calabria in BOLD (ID: HYMAA051-18, HYMAA052-18) is 100% and 99.61%.

#### Andrena ampla

Warncke, 1967

0EE12A36-B06F-5477-BFFD-0D947ECA09C4

##### Materials

- **Type status:** Other material. **Occurrence:** recordedBy: Sirio Gamba; sex: 1 female; occurrenceID: 43B89F36-F147-5E6B-A081-0A81F145BCEB; **Location:** countryCode: IT; stateProvince: Liguria; county: Imperia; municipality: Pigna; verbatimElevation: 1592 m; verbatimLatitude: 43.9925; verbatimLongitude: 7.6738; **Identification:** identifiedBy: Maurizio Cornalba, Paolo Biella; **Event:** eventDate: 2022-06-02; eventRemarks: on *Saxifragacallosa*; **Record Level:** collectionCode: SGC
- **Type status:** Other material. **Occurrence:** recordedBy: Christophe Praz, Dimitri Bénon; sex: 1 female; occurrenceID: 35DF6A0B-394E-555E-8575-FE315571E544; **Location:** countryCode: IT; stateProvince: Piemonte; county: Torino; municipality: Cesana Torinese; verbatimLatitude: 44.95001; verbatimLongitude: 6.80161; **Identification:** identifiedBy: Christophe Praz; **Event:** eventDate: 2022-07-07; **Record Level:** collectionCode: CPC

##### Notes

The record from Piemonte was communicated to us by Christophe Praz. *A.ampla* had been previously reported in Italy only from Valle d’Aosta (Schmid-Egger 2005). The present records are the first from Liguria and Piemonte. They suggest that *A.ampla* may occur all along the Italian side of the Southwestern Alps. The Ligurian specimen has been confirmed by DNA barcoding: match with reference sequences between 100% and 98.86% (mean: 99.81%).

#### Andrena binominata

Smith, 1853

93C50D98-225C-572D-99F9-C0E0B120AC91

##### Materials

- **Type status:** Other material. **Occurrence:** recordedBy: Rosa Ranalli; sex: 2 females; occurrenceID: 11BAE865-6DE4-5CF0-8847-124AA5543FC7; **Location:** countryCode: IT; stateProvince: Puglia; county: Bari; municipality: Polignano a Mare; verbatimLatitude: 41.0008; verbatimLongitude: 17.1919; **Identification:** identifiedBy: Simone Flaminio; **Event:** eventDate: 2022-02-12; **Record Level:** collectionCode: SFC

##### Notes

This appears to be the first record of *A.binominata* from continental Italy. Previously known in Italy only from Sicilia (Aubert et al. 2010; Fig. 1B).

#### Andrena bucephala

Stephens, 1846

6D43535D-1AE1-56D3-BC5A-515C0737B9EE

##### Materials

- **Type status:** Other material. **Occurrence:** recordedBy: Marco Selis; sex: 1 female; occurrenceID: AD5FF8DF-146C-59D6-87A8-9AA4A55C5477; **Location:** countryCode: IT; stateProvince: Lazio; county: Viterbo; municipality: Viterbo; locality: Necropoli etrusca di Norchia; verbatimElevation: 130 m; verbatimLatitude: 42.3383; verbatimLongitude: 11.9478; **Identification:** identifiedBy: Thomas J. Wood; **Event:** eventDate: 2021-04-23; **Record Level:** collectionCode: MSC

##### Notes

Our find represents a very significant range extension for this species, which was previously known in Italy only from the Po Plain (Gusenleitner and Schwarz 2002: 988).

#### Andrena compta

Lepeletier, 1841

6275019A-6244-5547-AAB3-95801CDCA37D

##### Materials

- **Type status:** Other material. **Occurrence:** recordedBy: Simone Flaminio; sex: 1 male; occurrenceID: D23561EC-D8CB-510C-898F-3E99B6179A1C; **Location:** countryCode: IT; stateProvince: Calabria; county: Reggio Calabria; municipality: Grotteria; verbatimElevation: 130 m; verbatimLatitude: 38.3445; verbatimLongitude: 16.2696; **Identification:** identifiedBy: Thomas J. Wood; **Event:** eventDate: 2022-04-23; **Record Level:** collectionCode: SFC

##### Notes

In Italy, previously known only from Sicilia and Sardegna (Comba 2019 and references therein). This is the first record for mainland Italy (Fig. 1A).

#### Andrena confinis

Stoeckhert, 1930

AEA7BD35-5466-512F-8182-F40FF7C3992F

##### Materials

- **Type status:** Other material. **Occurrence:** recordedBy: Maurizio Cornalba; sex: 1 male; occurrenceID: 8F313DBC-14BA-548B-B03D-B88CFFA1979C; **Location:** countryCode: IT; stateProvince: Lombardia; county: Pavia; municipality: Cecima; verbatimElevation: 666 m; verbatimLatitude: 44.8247; verbatimLongitude: 9.0767; **Identification:** identifiedBy: Maurizio Cornalba; **Event:** eventDate: 2022-04-08; **Record Level:** collectionCode: MCC
- **Type status:** Other material. **Occurrence:** recordedBy: Maurizio Cornalba; sex: 1 male; occurrenceID: 7B6CF6F8-E753-51E4-BBCF-E048481AC418; **Location:** countryCode: IT; stateProvince: Lombardia; county: Pavia; municipality: Cecima; verbatimElevation: 651 m; verbatimLatitude: 44.8257; verbatimLongitude: 9.0753; **Identification:** identifiedBy: Maurizio Cornalba; **Event:** eventDate: 2023-03-28; **Record Level:** collectionCode: MCC

##### Notes

Found in patrolling flight along paths in deciduous woodland. One specimen confirmed by DNA barcoding: 100% match with all *A.confinis* sequences in BOLD. These appear to be the first Italian records of the species. However, as *A.confinis* was formerly considered conspecific with *Andrenacongruens* Schmiedeknecht, 1883, specimens of *A.confinis* from Italy are almost certainly present in collections under the latter name. *A.confinis* is said to occur in Italy by Scheuchl and Willner (2016). However, this statement does not seem to be based on actual records, but only on inference. In fact, they mention only records from England, several Central European countries, Bashkortostan in Russia and Greece and then comment that the species almost certainly occurs also in the areas in between.

#### Andrena nigroviridula

Dours, 1873

820CC711-F626-5798-BF17-DEB2A133B9E0

##### Materials

- **Type status:** Other material. **Occurrence:** recordedBy: Simone Flaminio; sex: 2 females; occurrenceID: FE60A6F3-4DDC-5675-B36F-8E10D1C7C59C; **Location:** countryCode: IT; stateProvince: Calabria; county: Reggio Calabria; municipality: Caulonia; verbatimLatitude: 38.338; verbatimLongitude: 16.4605; **Identification:** identifiedBy: Thomas J. Wood; **Event:** eventDate: 2022-04-23; **Record Level:** collectionCode: SFC, MZUR
- **Type status:** Other material. **Occurrence:** recordedBy: Marco Selis; sex: 1 male, 1 female; occurrenceID: 97E59920-0AAC-5B2D-B7F3-3AB0C31AC7AC; **Location:** countryCode: IT; stateProvince: Umbria; county: Terni; municipality: Attigliano; locality: fiume Tevere; verbatimElevation: 50 m; verbatimLatitude: 42.5087; verbatimLongitude: 12.2792; **Identification:** identifiedBy: Marco Selis; **Event:** eventDate: 2023-03-17/22; **Record Level:** collectionCode: MSC

##### Notes

Known from Sicilia (Warncke 1967). Specimens from Lazio are mentioned by Comba (2019). The couple from Umbria were collected on *Sinapis sp*.

#### Andrena semilaevis

Pérez, 1903

63F162D7-F089-58E1-8BF2-D84C0D38FCEA

##### Materials

- **Type status:** Other material. **Occurrence:** recordedBy: Marco Selis; sex: 1 female; occurrenceID: 0ADC106E-404C-5E43-9DDF-C3DA4F373444; **Location:** countryCode: IT; stateProvince: Lazio; county: Rieti; municipality: Lonessa; locality: Sella di Leonessa; verbatimElevation: 1700-1870 m; verbatimLatitude: 42.48; verbatimLongitude: 13.0075; **Identification:** identifiedBy: Marco Selis, vid. T.J. Wood; **Event:** eventDate: 2021-07-04; **Record Level:** collectionCode: MSC
- **Type status:** Other material. **Occurrence:** recordedBy: Marco Selis; sex: 3 females; occurrenceID: D5AF1CDE-03C8-5F7B-BBC1-D3B7D74C3DEF; **Location:** countryCode: IT; stateProvince: Lazio; county: Rieti; municipality: Lonessa; locality: Sella di Leonessa; verbatimElevation: 1700-1870 m; verbatimLatitude: 42.48; verbatimLongitude: 13.0075; **Identification:** identifiedBy: Marco Selis, vid. T.J. Wood; **Event:** eventDate: 2022-06-19; **Record Level:** collectionCode: MSC

##### Notes

One specimen confirmed by DNA barcoding: 99.85-98.92% match (mean: 99.65%) with the *A.semilaevis* sequences in BOLD. In Italy, previously known from the Alpine regions and Toscana (Comba 2019 and references therein).

#### Halictus carinthiacus

Blüthgen, 1936

9BE24353-D725-5B3B-A84F-7DD6C1E49092

##### Materials

- **Type status:** Other material. **Occurrence:** recordedBy: Elena Gazzea; sex: 1 male; occurrenceID: 5EF47310-9CB2-5010-8BFA-145117274DFD; **Location:** countryCode: IT; stateProvince: Veneto; county: Belluno; municipality: Belluno; locality: Nevegal; verbatimElevation: 1200 m; verbatimLatitude: 46.0997; verbatimLongitude: 12.2995; **Identification:** identifiedBy: Maurizio Mei, Andree Cappellari; **Event:** eventDate: 2021-08-17/30; **Record Level:** collectionCode: MZUR

##### Notes

A rare species, previously known in Italy from Liguria, Lombardia and Friuli Venezia Giulia with isolated records (Ebmer 1988). Images: Suppl. material 2, Figs. S1 and S2.

#### Lasioglossum algericolellum

(Strand, 1909)

8C8644FB-A106-546C-BC9A-7882A92B7242

##### Materials

- **Type status:** Other material. **Occurrence:** recordedBy: Simone Flaminio; sex: 6 females; occurrenceID: 61E8C0E9-75EA-53AA-A47A-DD0C96DFE0CB; **Location:** countryCode: IT; stateProvince: Sicilia; county: Trapani; municipality: Pantelleria; verbatimLatitude: 36.8169; verbatimLongitude: 11.9297; **Identification:** identifiedBy: Simone Flaminio; **Event:** eventDate: 2022-05-16; **Record Level:** collectionCode: SFC

##### Notes

*L.algericolellum* was synonymised with *L.pauxillum* (Schenck, 1853) by Blüthgen (1922), but it was resurrected as a valid species by Ortiz-Sánchez and Pauly (2017). Sicilian records of *L.pauxillum* should be checked for the possible presence of further specimens of *L.algericolellum*.

#### Lasioglossum monstrificum

(Morawitz, 1891)

08E72A5D-03FC-578C-9C3A-4C2B4FEEEB12

##### Materials

- **Type status:** Other material. **Occurrence:** recordedBy: Simone Flaminio; sex: 3 females; occurrenceID: 4AE4FCCC-B125-53F8-BEFE-3C8659031413; **Location:** countryCode: IT; stateProvince: Emilia-Romagna; county: Bologna; municipality: Bologna; verbatimLatitude: 44.4506; verbatimLongitude: 11.3675; **Identification:** identifiedBy: Simone Flaminio; **Event:** eventDate: 2019-04-24; **Record Level:** collectionCode: SFC
- **Type status:** Other material. **Occurrence:** recordedBy: Simone Flaminio; sex: 1 female; occurrenceID: 83423807-6BFE-561C-8CC3-0A6613D8151E; **Location:** countryCode: IT; stateProvince: Emilia-Romagna; county: Bologna; municipality: Bologna; verbatimLatitude: 44.4506; verbatimLongitude: 11.3675; **Identification:** identifiedBy: Simone Flaminio; **Event:** eventDate: 2019-06-01; **Record Level:** collectionCode: SFC
- **Type status:** Other material. **Occurrence:** recordedBy: Simone Flaminio; sex: 1 female; occurrenceID: B540D20D-39B3-51DE-92C9-4B4FC39371BB; **Location:** countryCode: IT; stateProvince: Emilia-Romagna; county: Bologna; municipality: Bologna; verbatimLatitude: 44.4506; verbatimLongitude: 11.3675; **Identification:** identifiedBy: Simone Flaminio; **Event:** eventDate: 2020-05-04; **Record Level:** collectionCode: SFC
- **Type status:** Other material. **Occurrence:** recordedBy: Simone Flaminio; sex: 1 male; occurrenceID: AA57ED6F-7989-5B06-924B-8210999711E1; **Location:** countryCode: IT; stateProvince: Emilia-Romagna; county: Bologna; municipality: Bologna; verbatimLatitude: 44.4506; verbatimLongitude: 11.3675; **Identification:** identifiedBy: Simone Flaminio; **Event:** eventDate: 2020-07-21; **Record Level:** collectionCode: SFC
- **Type status:** Other material. **Occurrence:** recordedBy: Marco Selis; sex: 1 male; occurrenceID: 24BC58BA-72FF-5BD1-B95E-6FD9915BC2FC; **Location:** countryCode: IT; stateProvince: Lazio; county: Viterbo; municipality: Bomarzo; locality: fiume Tevere; verbatimElevation: 50 m; verbatimLatitude: 42.5131; verbatimLongitude: 12.2744; **Identification:** identifiedBy: Marco Selis; **Event:** eventDate: 2022-07-19; **Record Level:** collectionCode: MSC

##### Notes

The distribution of *L.monstrificum* (= *Halictussabulosus* Warncke, 1986) in Italy is unclear, due in part to past confusion with *L.sexstrigatum* (Schenck, 1870).

#### Lasioglossum strictifrons

(Vachal, 1895)

DB2C3520-C1F0-5184-A0A8-354DA44FDAA4

##### Materials

- **Type status:** Other material. **Occurrence:** recordedBy: Marco Selis; sex: 1 female; occurrenceID: 587B37EF-AED9-5D58-A34E-8568D2D91216; **Location:** countryCode: IT; stateProvince: Lazio; county: Viterbo; municipality: Bomarzo; locality: fiume Tevere; verbatimElevation: 50 m; verbatimLatitude: 42.5131; verbatimLongitude: 12.2744; **Identification:** identifiedBy: Marco Selis; **Event:** eventDate: 2022-06-28; **Record Level:** collectionCode: MSC
- **Type status:** Other material. **Occurrence:** recordedBy: Marco Selis; sex: 3 males, 4 females; occurrenceID: 9D7A095A-D57C-538F-A283-7A28D63D43ED; **Location:** countryCode: IT; stateProvince: Lazio; county: Viterbo; municipality: Bomarzo; locality: fiume Tevere; verbatimElevation: 50 m; verbatimLatitude: 42.5131; verbatimLongitude: 12.2744; **Identification:** identifiedBy: Marco Selis; **Event:** eventDate: 2022-07-02; **Record Level:** collectionCode: MSC
- **Type status:** Other material. **Occurrence:** recordedBy: Marco Selis; sex: 3 males, 5 females; occurrenceID: C57498DD-0C68-5A30-A189-B2DC0232D336; **Location:** countryCode: IT; stateProvince: Lazio; county: Viterbo; municipality: Bomarzo; locality: fiume Tevere; verbatimElevation: 50 m; verbatimLatitude: 42.5131; verbatimLongitude: 12.2744; **Identification:** identifiedBy: Marco Selis; **Event:** eventDate: 2022-07-05; **Record Level:** collectionCode: MSC, SFC, MIB:ZPL
- **Type status:** Other material. **Occurrence:** recordedBy: Marco Selis; sex: 1 male, 2 females; occurrenceID: E9D9A083-2ECD-5108-BF3E-293FB67A80CB; **Location:** countryCode: IT; stateProvince: Lazio; county: Viterbo; municipality: Bomarzo; locality: fiume Tevere; verbatimElevation: 50 m; verbatimLatitude: 42.5131; verbatimLongitude: 12.2744; **Identification:** identifiedBy: Marco Selis; **Event:** eventDate: 2022-07-10; **Record Level:** collectionCode: MSC
- **Type status:** Other material. **Occurrence:** recordedBy: Marco Selis; sex: 1 male; occurrenceID: BFA155C0-9DDA-5453-892B-B8BB306C138F; **Location:** countryCode: IT; stateProvince: Lazio; county: Viterbo; municipality: Bomarzo; locality: fiume Tevere; verbatimElevation: 50 m; verbatimLatitude: 42.5131; verbatimLongitude: 12.2744; **Identification:** identifiedBy: Marco Selis; **Event:** eventDate: 2022-07-19; **Record Level:** collectionCode: MSC

##### Notes

**Figure 3.**
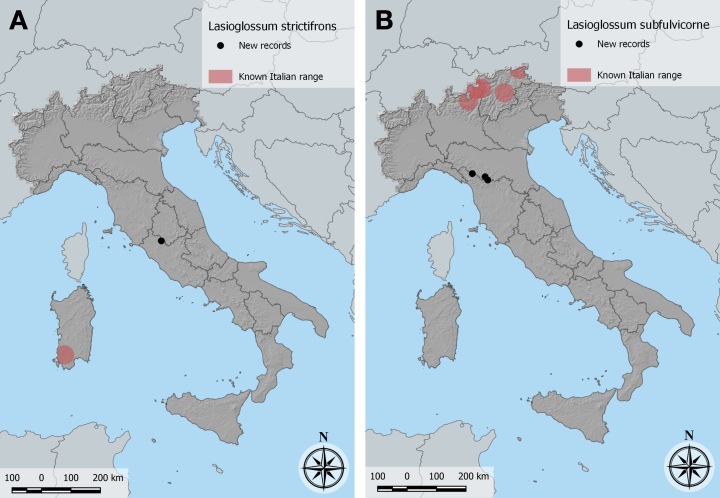
Known Italian range and new records of (A) *Lasioglossumstrictifrons* (Vachal, 1895) and (B) *Lasioglossumsubfulvicorne* (Blüthgen, 1934).

**Figure 4.**
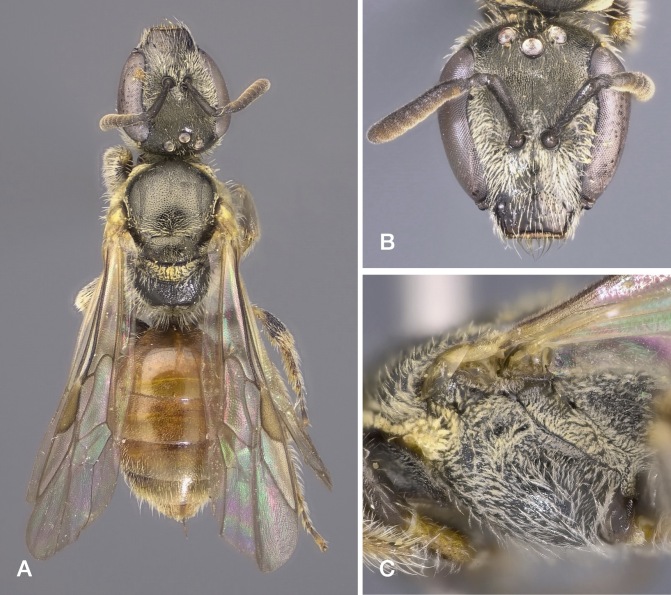
*Lasioglossumstrictifrons* (Vachal, 1895), female from Bomarzo, (A) habitus; (B) face; (C) pleura.

All specimens were collected on *Lythrumsalicaria*, on the banks of the River Tiber. Our records extend the range of the species to continental Italy (Fig. 3A). Previously known in Italy from a single record from south-western Sardegna (Ebmer 1986). Elsewhere, the species range includes Iberia, southern France and the Maghreb (Ebmer 1986). We sequenced two of our specimens. Only one sequence, labelled *Lasioglossumstrictifrons*, from southern Portugal, was available in BOLD for comparison, matched at 99.02 and 99.16% by our sequences. Matches with BOLD sequences labelled as other species were all below 92%. Images: Fig. 4.

#### Lasioglossum subaenescens

(Pérez, 1896)

67A14C53-A264-547B-89BB-DCC668A309E6

##### Materials

- **Type status:** Other material. **Occurrence:** recordedBy: Marco Selis; sex: 1 female; occurrenceID: 4926CF22-DE19-52C1-8450-0752FD3A5801; **Location:** countryCode: IT; stateProvince: Lazio; county: Viterbo; municipality: Bomarzo; locality: fiume Tevere; verbatimElevation: 50 m; verbatimLatitude: 42.5131; verbatimLongitude: 12.2744; **Identification:** identifiedBy: Marco Selis, Simone Flaminio; **Event:** eventDate: 2022-07-02; **Record Level:** collectionCode: MSC

##### Notes

The only published record of *L.subaenescens* from Italy known to us is that of two females from Molise, one of them determined by Ebmer (Quaranta et al. 2004).

#### Lasioglossum subfulvicorne

(Blüthgen, 1934)

A3BF8511-4FDC-5418-8F5C-CB796A59A511

##### Materials

- **Type status:** Other material. **Occurrence:** recordedBy: Marco Selis; sex: 3 males; occurrenceID: F2FB9840-0C84-5817-AAC1-82F6B56AE8F9; **Location:** countryCode: IT; stateProvince: Emilia-Romagna; county: Modena; municipality: Sestola; locality: Monte Cimone; verbatimElevation: 1500-1800 m; verbatimLatitude: 44.2083; verbatimLongitude: 10.7172; **Identification:** identifiedBy: Marco Selis; **Event:** eventDate: 2021-08-15; **Record Level:** collectionCode: MSC
- **Type status:** Other material. **Occurrence:** recordedBy: Marco Selis; sex: 1 male, 2 females; occurrenceID: 396AE5B8-C76E-5352-9744-2C5D96B538C6; **Location:** countryCode: IT; stateProvince: Emilia-Romagna; county: Bologna; municipality: Lizzano in Belvedere; locality: Corno alle Scale, Lago Scaffaiolo; verbatimElevation: 1500-1800 m; verbatimLatitude: 44.1221; verbatimLongitude: 10.8136; **Identification:** identifiedBy: Marco Selis; **Event:** eventDate: 2021-08-10; **Record Level:** collectionCode: MSC
- **Type status:** Other material. **Occurrence:** recordedBy: M. Violi; sex: 1 female; occurrenceID: 6DD5B38A-CFE0-5C4B-B4E1-CDD1D97FBFFE; **Location:** countryCode: IT; stateProvince: Emilia-Romagna; county: Reggio Emilia; municipality: Cerreto Laghi; locality: between Lago Cerretano and Monte la Nuda; verbatimElevation: 1350-1893 m; verbatimLatitude: 44.2883; verbatimLongitude: 10.2408; **Identification:** identifiedBy: Marco Selis; **Event:** eventDate: 2021-08-14; **Record Level:** collectionCode: MSC
- **Type status:** Other material. **Occurrence:** recordedBy: M. Violi; sex: 1 male; occurrenceID: FCF77CAA-1CDE-51B6-A898-4A391FF7781E; **Location:** countryCode: IT; stateProvince: Emilia-Romagna; county: Reggio Emilia; municipality: Cerreto Laghi; locality: between Lago Cerretano and Monte la Nuda hut; verbatimElevation: 1350-1670 m; verbatimLatitude: 44.2922; verbatimLongitude: 10.2411; **Identification:** identifiedBy: Marco Selis; **Event:** eventDate: 2021-08-07; **Record Level:** collectionCode: MSC

##### Notes

Two specimens were confirmed by DNA barcoding with sequences matching reference sequences between 99.34% and 99.01% (mean 99.10%, excluding sequences labelled “subfulvicorne” appearing as single representatives with this name in clades dominated by other species in the neighbour-joining tree). The species had been reported in Italy only from the Alps in Trentino-Alto Adige/Südtirol (Ebmer 1988) and Lombardia (Praz et al. 2022), so our records appear to be the first for the Apennines (Fig. 2B).

#### Seladonia gavarnica

(Pérez, 1903)

D24431A3-5BEC-5274-8689-BB050B7C8340

##### Materials

- **Type status:** Other material. **Occurrence:** recordedBy: Marco Selis; sex: 1 female; occurrenceID: 72B707D1-0C63-5908-93F5-6964A2B7DE8F; **Location:** countryCode: IT; stateProvince: Lazio; county: Rieti; municipality: Lonessa; locality: Sella di Leonessa; verbatimElevation: 1700-1870 m; verbatimLatitude: 42.48; verbatimLongitude: 13.0075; **Identification:** identifiedBy: Marco Selis; **Event:** eventDate: 2021-08-22; **Record Level:** collectionCode: MSC
- **Type status:** Other material. **Occurrence:** recordedBy: Marco Selis; sex: 5 males, 10 females; occurrenceID: 0588BE7B-C83F-5A93-AB6F-F7F13FA1DFD4; **Location:** countryCode: IT; stateProvince: Lazio; county: Rieti; municipality: Lonessa; locality: Sella di Leonessa; verbatimElevation: 1700-1870 m; verbatimLatitude: 42.48; verbatimLongitude: 13.0075; **Identification:** identifiedBy: Marco Selis; **Event:** eventDate: 2022-08-04; **Record Level:** collectionCode: MSC

##### Notes

In Italy, *S.gavarnica* has been recorded in the Sibillini Mountains (Piatti and Ricciardelli D'Albore 2006). There is also an indication of its occurrence in Piemonte (Comba 2019). Sequencing of one of our specimens yielded a 658 bp sequence matching the single one in BOLD labelled *Halictusgavarnicus*, from the French Maritime Alps, at 99.39%. All matches with other BOLD sequences were well below 96.5%.

#### Macropis europaea

Warncke, 1973

E0BF8CDB-85A0-5101-A721-ECEAE26AC03F

##### Materials

- **Type status:** Other material. **Occurrence:** recordedBy: Maurizio Mei; sex: 8 males, 6 females; occurrenceID: 1451BEBA-0F0F-5765-9D9F-EED71EA08897; **Location:** countryCode: IT; stateProvince: Lazio; county: Roma; municipality: Anticoli Corrado; verbatimElevation: 320 m; verbatimLatitude: 42.0185; verbatimLongitude: 12.9929; **Identification:** identifiedBy: Maurizio Mei; **Event:** eventDate: 2021-07-16; **Record Level:** collectionCode: MEC, MZUR
- **Type status:** Other material. **Occurrence:** recordedBy: Marco Selis; sex: 2 males, 8 females; occurrenceID: 46CD601D-ACE2-54F2-BDF8-D237976FCA62; **Location:** countryCode: IT; stateProvince: Lazio; county: Viterbo; municipality: Bomarzo; locality: fiume Tevere; verbatimElevation: 50 m; verbatimLatitude: 42.5131; verbatimLongitude: 12.2744; **Identification:** identifiedBy: Marco Selis; **Event:** eventDate: 2022-07-02; **Record Level:** collectionCode: MSC
- **Type status:** Other material. **Occurrence:** recordedBy: Marco Selis; sex: 3 females; occurrenceID: 0F3213A8-A91A-5108-9B08-25DA4A93526C; **Location:** countryCode: IT; stateProvince: Lazio; county: Viterbo; municipality: Bomarzo; locality: fiume Tevere; verbatimElevation: 50 m; verbatimLatitude: 42.5131; verbatimLongitude: 12.2744; **Identification:** identifiedBy: Marco Selis; **Event:** eventDate: 2022-07-05; **Record Level:** collectionCode: MSC

##### Notes

On flowers of *Lysimachiavulgaris*, in clearings along the banks of the Rivers Aniene and Tiber. Our records significantly extend the range of the species, which had previously been recorded in Italy only in the north (Comba 2019 and references therein).

#### Anthidiellum breviusculum

(Pérez, 1890)

404D63A2-F1EF-5F26-ABFD-4F56D352D3A5

##### Materials

- **Type status:** Other material. **Occurrence:** recordedBy: Christophe Praz, Gilles Carron; sex: 1 male, 1 female; occurrenceID: 93BE74A9-D1EF-501E-A80F-FD591C253D2E; **Location:** countryCode: IT; stateProvince: Piemonte; county: Torino; municipality: Salbertrand; locality: Fenil; **Identification:** identifiedBy: Christophe Praz; **Event:** eventDate: 2006-07-01; eventRemarks: on *Teucrium* sp.; **Record Level:** collectionCode: CPC
- **Type status:** Other material. **Occurrence:** recordedBy: Christophe Praz, Dimitri Bénon; sex: 1 male, 1 female; occurrenceID: 3F6C2B48-E93C-5DEE-990A-7CA2C3A4C522; **Location:** countryCode: IT; stateProvince: Piemonte; county: Torino; municipality: Mompantero; verbatimLatitude: 45.1454; verbatimLongitude: 7.0806; **Identification:** identifiedBy: Christophe Praz; **Event:** eventDate: 2022-07-07; **Record Level:** collectionCode: CPC

##### Notes

These records were communicated to us by Christophe Praz. *Anthidiellumbreviusculum* s.l. is split by Kasparek et al. (2023) into three species, *A.breviusculum* s.s. from Iberia and France, *A.africanum* Kasparek, 2023 from the Maghreb and *A.troodicum* (Mavromoustakis, 1949) which ranges from the Aegean through Anatolia and the Levant to Iran. *A.breviusculum* is here first reported from Italy. Its occurrence close to the French border is not unexpected, given its presence in the Var and Roia drainages just across the border (Warncke 1980, Schmid-Egger 2011).

#### Chelostoma grande

(Nylander, 1852)

B05AEB5C-2B6D-5D25-BBAF-70D8C4A3DD5F

##### Materials

- **Type status:** Other material. **Occurrence:** recordedBy: Sirio Gamba; sex: 1 male; occurrenceID: 1FAB12A8-6B2B-54AF-ADCD-F86B18BBF132; **Location:** countryCode: IT; stateProvince: Liguria; county: Imperia; municipality: Rocchetta Nervina; verbatimElevation: 1155 m; verbatimLatitude: 43.9411; verbatimLongitude: 7.5886; **Identification:** identifiedBy: Sirio Gamba; **Event:** eventDate: 2020-06-06; **Record Level:** collectionCode: SGC
- **Type status:** Other material. **Occurrence:** recordedBy: Christophe Praz, Dimitri Bénon; sex: 1 female; occurrenceID: F87C6B24-DB3A-589A-A575-A073BE3CC456; **Location:** countryCode: IT; stateProvince: Piemonte; county: Torino; municipality: Cesana Torinese; verbatimLatitude: 44.95001; verbatimLongitude: 6.80161; **Identification:** identifiedBy: Christophe Praz; **Event:** eventDate: 2022-07-07; **Record Level:** collectionCode: CPC

##### Notes

The record from Piemonte was communicated to us by Christophe Praz. The Ligurian specimen was found in patrolling flight along a path in woodland. To our knowledge, these are only the third and fourth records of *C.grande* in Italy. The previous records are from Val di Fiemme in Trentino-Alto Adige/Südtirol (Cobelli 1903) and from the upper Val di Susa in Piemonte (Westrich 1993). Images: Suppl. material 2, Figs. S3 and S4.

#### Coelioxys alatus

Foerster, 1853

0345F779-03AE-5D2E-81DC-4C6CDB75207D

##### Materials

- **Type status:** Other material. **Occurrence:** recordedBy: Elena Gazzea; sex: 1 female; occurrenceID: BCE7F4EA-DA44-5A3B-BAB9-65C20C6525AE; **Location:** countryCode: IT; stateProvince: Veneto; county: Belluno; municipality: Voltago Agordino; verbatimElevation: 990 m; verbatimLatitude: 46.2662; verbatimLongitude: 11.9975; **Identification:** identifiedBy: Maurizio Mei, Andree Cappellari; **Event:** eventDate: 2021-08-10/22; **Record Level:** collectionCode: MZUR

##### Notes

**Figure 5.**
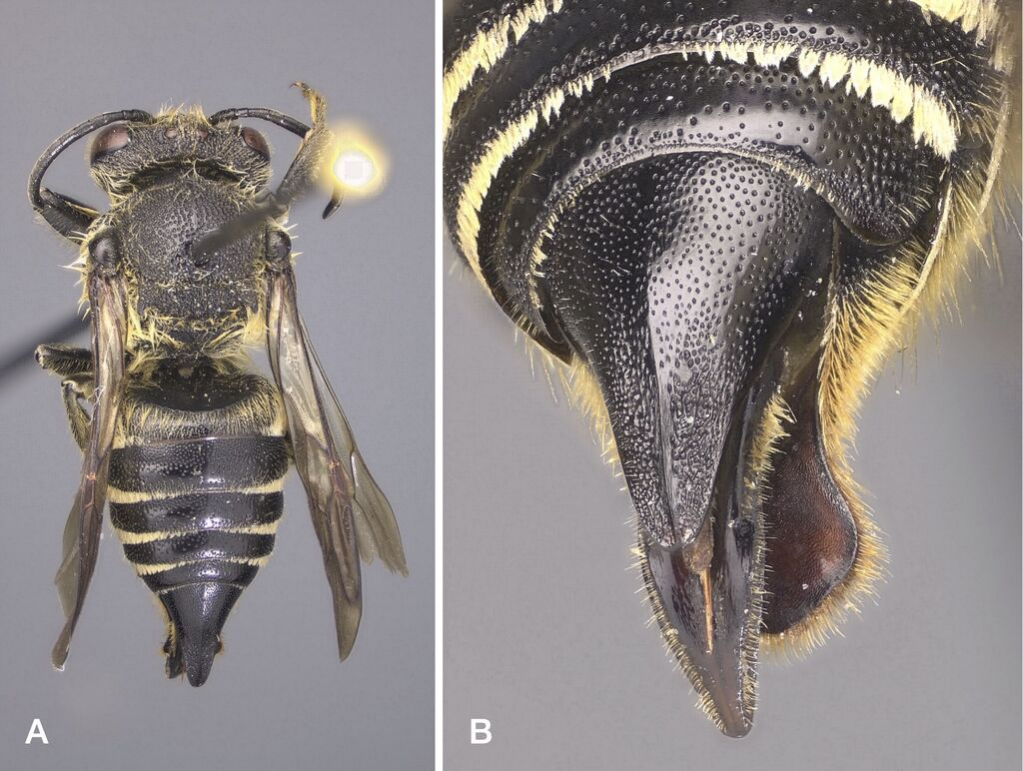
*Coelioxysalatus* Foerster, 1853 female from Voltago Agordino, (A) habitus; (B) end tergites and sternites.

Caught in a pan trap in an area with a significant presence of *Megachileligniseca* (Kirby, 1802), the main host of the species. This is the first confirmed record of *C.alatus* for Italy. Discover Life (Ascher and Pickering 2023) indicates the presence of the species in Italy, without details. It is not clear what this claim is based on. A possible source is Stöckl (2000), where *C.alatus* is mentioned as (doubtfully) occurring in Südtirol, based on Biegeleben (1929). However, Biegeleben's paper, which is a general exposition of parasitism in bees, does not contain records of *C.alatus* or, for that matter, of any bee species. The only place where *C.alatus* is mentioned is a list of host bees and their parasites in the last two pages of the paper, preceded by a sentence which, in English translation, reads: "We owe to Prof. Dr. Bischoff, who excels in the study of the biology of Hymenoptera, a detailed table of the different species of host bees and their parasites. Some of them, namely the ones that are most widespread in our region, are listed here according to this table". In the case at hand, "widespread" likely does not refer to *C.alatus*, which is rare everywhere in Europe (Devalez 2010), but to its host *Megachileligniseca* which is ubiquitous, though uncommon, in Südtirol. For good measure, we inspected the Biegeleben collection, now housed in the Museo Civico di Zoologia in Rome, but could not locate any specimens of *C.alatus*. Images: Fig. 5.

#### Megachile lapponica

Thomson, 1872

B5C11DF4-C527-5415-919F-C46C8E02AF14

##### Materials

- **Type status:** Other material. **Occurrence:** recordedBy: Elena Gazzea; sex: 1 female; occurrenceID: C6974FF3-F5F4-57D9-8AA6-EFB07497BF37; **Location:** countryCode: IT; stateProvince: Veneto; county: Belluno; municipality: Livinallongo; locality: Cherz; verbatimElevation: 1780 m; verbatimLatitude: 46.5089; verbatimLongitude: 11.9118; **Identification:** identifiedBy: Maurizio Mei, Andree Cappellari, vid. C. Praz; **Event:** eventDate: 2021-07-28/08-10; **Record Level:** collectionCode: MZUR
- **Type status:** Other material. **Occurrence:** recordedBy: Marco Bonifacino; sex: 1 male, 1 female; occurrenceID: 7889BBF0-C297-58D8-BF91-03EE1C0B8419; **Location:** countryCode: IT; stateProvince: Abruzzo; county: L'Aquila; municipality: Lucoli; locality: Valle Leona; verbatimElevation: 1685 m; verbatimLatitude: 42.2059; verbatimLongitude: 13.4202; **Identification:** identifiedBy: Marco Bonifacino; **Event:** eventDate: 2021-07-06; **Record Level:** collectionCode: MBC

##### Notes

**Figure 6.**
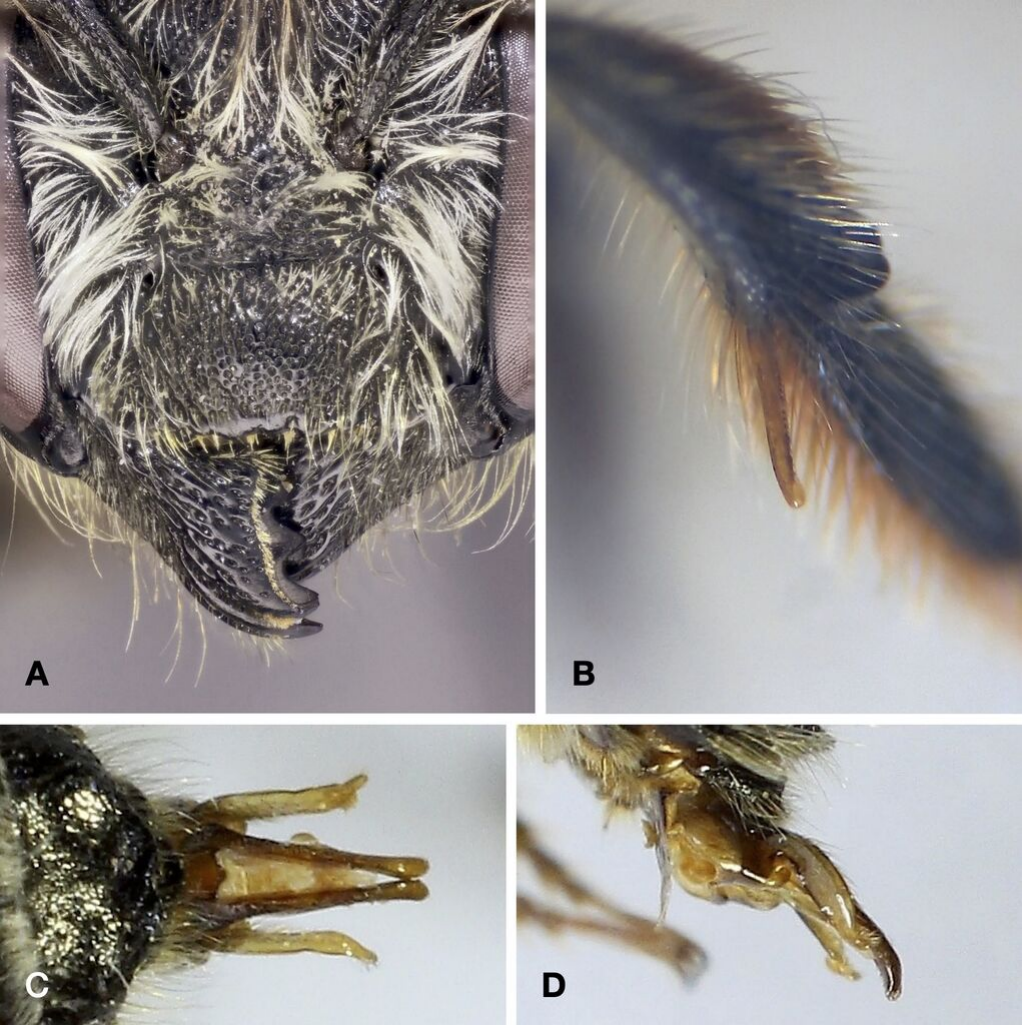
*Megachilelapponica* Thomson, 1872, (A) female from Livinallongo, clypeus and mandibles; (B) female from Lucoli, inner hind tibial spur; (C), (D) male from Lucoli, genitalia.

The specimen from Veneto was caught in a pan trap and the ones from Abruzzo on *Epilobium* sp, in agreement with the stated oligolecty of *M.lapponica* on *Epilobium* (Amiet et al. 2004, Scheuchl 2006). These are the first records of the species from Italy. The one from Abruzzo is particularly noteworthy because, excepting isolated records from Greece, it is by far the most southerly in Europe. Even in the Alps, the records of *M.lapponica* are sporadic, although this may be due more to the difficulty of intercepting the species than to its actual rarity (C. Praz, personal communication 2022). Images: Fig. 6.

#### Megachile opacifrons

Pérez, 1897

C966FA46-9886-505A-85F7-EA00709E6702

##### Materials

- **Type status:** Other material. **Occurrence:** recordedBy: Sirio Gamba; sex: 1 male; occurrenceID: ACF18215-FD76-5D51-BF2B-DADA9FB21E0C; **Location:** countryCode: IT; stateProvince: Liguria; county: Imperia; municipality: Apricale; verbatimElevation: 305 m; verbatimLatitude: 43.873; verbatimLongitude: 7.6683; **Identification:** identifiedBy: Maurizio Cornalba, vid. C. Praz; **Event:** eventDate: 2021-07-18; **Record Level:** collectionCode: SGC

##### Notes

**Figure 7.**
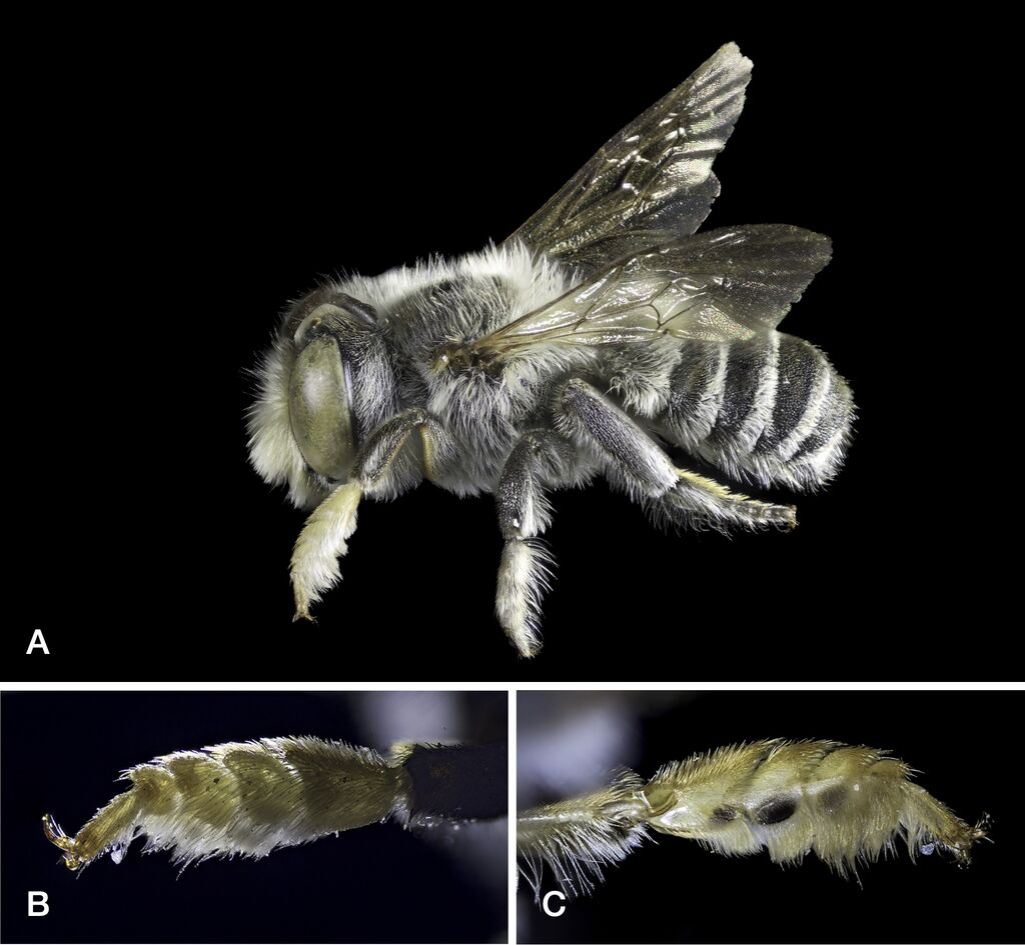
*Megachileopacifrons* Pérez, 1897, male from Apricale, (A) habitus; (B) fore tarsi, outer view; (C) fore tarsi, inner view.

This is the first Italian record of *M.opacifrons*. The specimen was found in a clearing surrounded by deciduous woodland, vineyards and an abandoned olive grove. *M.opacifrons* has a West Mediterranean distribution and was known to occur in southern France east to the upper Roia Valley (Schmid-Egger 2011). Hence, it was conceivable that it might also occur in neighbouring parts of Italy. Images: Fig. 7.

#### Megachile semicircularis

auct. nec Zanden, 1996

83032D31-E2E9-544D-A266-B13CFBB0FDE7

##### Materials

- **Type status:** Other material. **Occurrence:** recordedBy: Marco Selis; sex: 2 females; occurrenceID: A9354F52-B924-55D9-835C-CDD088556550; **Location:** countryCode: IT; stateProvince: Lazio; county: Viterbo; municipality: Viterbo; locality: Necropoli etrusca di Norchia; verbatimElevation: 130-160 m; verbatimLatitude: 42.3381; verbatimLongitude: 11.9456; **Identification:** identifiedBy: Christophe Praz; **Event:** eventDate: 2022-06-24; **Record Level:** collectionCode: MSC, CPC

##### Notes

This species is recorded for the first time in Italy. Elsewhere, it is distributed from Greece through Turkey and Crimea to Iran (Ascher and Pickering 2023, C. Praz, personal communication 2022). It has generally been known as *Megachilesemicircularis* Zanden. However, the holotype of the latter is a male of *M.apicalis* Spinola, 1808 (C. Praz, personal communication 2022).

#### Osmia heteracantha

Pérez, 1896

C1546C9A-FC18-58F2-BA23-7EC8D9E8CF79

##### Materials

- **Type status:** Other material. **Occurrence:** recordedBy: Maurizio Mei; sex: 3 females; occurrenceID: DD1C6386-BED4-53AC-92D2-9019E4522C79; **Location:** countryCode: IT; stateProvince: Sardegna; county: Oristano; municipality: San Giovanni di Sinis; verbatimElevation: 10 m; verbatimLatitude: 39.8697; verbatimLongitude: 8.4395; **Identification:** identifiedBy: Maurizio Mei; **Event:** eventDate: 2022-06-24/07-14; **Record Level:** collectionCode: MEC, MZUR
- **Type status:** Other material. **Occurrence:** recordedBy: Maurizio Mei; sex: 1 female; occurrenceID: 3FCA9E3C-35EC-53DD-9427-D548B7E17CF4; **Location:** countryCode: IT; stateProvince: Sardegna; county: Oristano; municipality: Oristano; locality: mouth of the river Tirso; verbatimElevation: 5 m; verbatimLatitude: 39.8878; verbatimLongitude: 8.5407; **Identification:** identifiedBy: Maurizio Mei; **Event:** eventDate: 2022-07-08; **Record Level:** collectionCode: MEC
- **Type status:** Other material. **Occurrence:** recordedBy: Pietro Niolu; sex: 1 female; occurrenceID: 9DF6C93F-3B99-5CE9-A496-2497692A0B0C; **Location:** countryCode: IT; stateProvince: Sardegna; county: Sassari; municipality: Villanova Monteleone; locality: Monte Aidos; verbatimLatitude: 40.444; verbatimLongitude: 8.4413; **Identification:** identifiedBy: Maurizio Mei; **Event:** eventDate: 2022-06-16; **Record Level:** collectionCode: CEUSS
- **Type status:** Other material. **Occurrence:** recordedBy: Pietro Niolu; sex: 1 female; occurrenceID: 4714A05E-C2B3-5C3B-BF96-93DF19854FA4; **Location:** countryCode: IT; stateProvince: Sardegna; county: Sassari; municipality: Villanova Monteleone; locality: Monte Aidos; verbatimLatitude: 40.444; verbatimLongitude: 8.4413; **Identification:** identifiedBy: Maurizio Mei; **Event:** eventDate: 2022-07-01; **Record Level:** collectionCode: CEUSS

##### Notes

*O.heteracantha* is first recorded from Sardegna. It was previously known to occur in Sicilia and mainland Italy (Müller 2022). Images: Suppl. material 2, Fig. S7.

#### Pseudoanthidium stigmaticorne

(Dours, 1873)

28EB221F-B6CE-50DC-866C-A1B40FA3FEF0

##### Materials

- **Type status:** Other material. **Occurrence:** recordedBy: Marco Selis; sex: 1 female; occurrenceID: BD721F71-8944-5A1F-B7BA-FE7B72C950A3; **Location:** countryCode: IT; stateProvince: Lazio; county: Viterbo; municipality: Viterbo; locality: Necropoli etrusca di Norchia; verbatimElevation: 130-160 m; verbatimLatitude: 42.3381; verbatimLongitude: 11.9456; **Identification:** identifiedBy: Marco Selis; **Event:** eventDate: 2019-06-01; **Record Level:** collectionCode: MSC
- **Type status:** Other material. **Occurrence:** recordedBy: Sirio Gamba; sex: 1 male, 2 females; occurrenceID: DDB2D9E4-0D27-5CC5-B2B4-E865BE2B259E; **Location:** countryCode: IT; stateProvince: Liguria; county: Imperia; municipality: San Biagio della Cima; verbatimElevation: 122 m; verbatimLatitude: 43.8225; verbatimLongitude: 7.655; **Identification:** identifiedBy: Sirio Gamba; **Event:** eventDate: 2020-05-09; **Record Level:** collectionCode: SGC, MIB:ZPL
- **Type status:** Other material. **Occurrence:** recordedBy: Sirio Gamba; sex: 2 males; occurrenceID: 935A4ADC-DFC5-520F-A815-4B6C2B61EA32; **Location:** countryCode: IT; stateProvince: Liguria; county: Imperia; municipality: Ventimiglia; verbatimElevation: 6 m; verbatimLatitude: 43.7877; verbatimLongitude: 7.6288; **Identification:** identifiedBy: Sirio Gamba; **Event:** eventDate: 2022-07-27; **Record Level:** collectionCode: SGC
- **Type status:** Other material. **Occurrence:** recordedBy: Sirio Gamba; sex: 1 male; occurrenceID: 1DE28E30-89F1-5421-BCBD-C78BADC2B9DD; **Location:** countryCode: IT; stateProvince: Liguria; county: Imperia; municipality: Camporosso; verbatimElevation: 32 m; verbatimLatitude: 43.8308; verbatimLongitude: 7.6318; **Identification:** identifiedBy: Sirio Gamba; **Event:** eventDate: 2022-07-28; **Record Level:** collectionCode: SGC

##### Notes

*P.stigmaticorne* was previously known in Italy only from Sicilia, Sardegna and Puglia (Litman et al. 2021). We sequenced two specimens from Liguria and one from Lazio, the resulting sequences matching reference sequences between 100% and 99.82% (mean 99.58%). The neighbour-joining tree unequivocally confirms the identifications.

#### Rhodanthidium siculum

(Spinola, 1838)

6C167220-386A-5E69-88C3-AF5C19B27C34

##### Materials

- **Type status:** Other material. **Occurrence:** recordedBy: Simone Flaminio; sex: 5 males, 3 females; occurrenceID: D453F8A2-E28A-5D68-9DEA-0494610D210E; **Location:** countryCode: IT; stateProvince: Calabria; county: Crotone; municipality: Cutro; verbatimLatitude: 38.9397; verbatimLongitude: 16.9622; **Identification:** identifiedBy: Simone Flaminio; **Event:** eventDate: 2022-04-21; **Record Level:** collectionCode: SFC

##### Notes

This appears to be the first record of *R.siculum* for continental Italy (Fig. 1B). The species was previously known in Italy only from Sicilia (Kasparek 2019).

#### Rhodanthidium sticticum

(Fabricius, 1787)

62113DFE-8ED1-5B7D-9D04-C262BA8C434B

##### Materials

- **Type status:** Other material. **Occurrence:** recordedBy: Marco Bonifacino; sex: 1 male; occurrenceID: B27F2E49-D719-5EBC-A604-331B06697EC0; **Location:** countryCode: IT; stateProvince: Liguria; county: Imperia; municipality: Ventimiglia; locality: Monte Grammondo; verbatimElevation: 650 m; verbatimLatitude: 43.8434; verbatimLongitude: 7.5294; **Identification:** identifiedBy: Marco Bonifacino; **Event:** eventDate: 2021-05-07; **Record Level:** collectionCode: MBC
- **Type status:** Other material. **Occurrence:** recordedBy: Marco Bonifacino; sex: 1 female; occurrenceID: 154D4860-271A-5B95-9143-28B50E472139; **Location:** countryCode: IT; stateProvince: Liguria; county: Imperia; municipality: Ventimiglia; locality: Colla di Bevera; verbatimElevation: 435 m; verbatimLatitude: 43.8294; verbatimLongitude: 7.5671; **Identification:** identifiedBy: Marco Bonifacino; **Event:** eventDate: 2020-06-18; **Record Level:** collectionCode: MBC
- **Type status:** Other material. **Occurrence:** recordedBy: Sirio Gamba; sex: 1 female; occurrenceID: 7500E0C6-A7D9-5933-BE7A-6B72EC4C9D32; **Location:** countryCode: IT; stateProvince: Liguria; county: Imperia; municipality: Perinaldo; verbatimElevation: 484 m; verbatimLatitude: 43.8666; verbatimLongitude: 7.6624; **Identification:** identifiedBy: Sirio Gamba; **Event:** eventDate: 2018-04-08; **Record Level:** collectionCode: SGC
- **Type status:** Other material. **Occurrence:** recordedBy: Sirio Gamba; sex: 1 male; occurrenceID: D107ECA6-41F7-5AD3-BDC3-95927C3D4BD6; **Location:** countryCode: IT; stateProvince: Liguria; county: Imperia; municipality: San Biagio della Cima; verbatimElevation: 338 m; verbatimLatitude: 43.8063; verbatimLongitude: 7.6419; **Identification:** identifiedBy: Sirio Gamba; **Event:** eventDate: 2019-04-14; **Record Level:** collectionCode: SGC
- **Type status:** Other material. **Occurrence:** recordedBy: Sirio Gamba; sex: 1 male; occurrenceID: 7C5903A4-0DA2-51E5-9A90-6A6ADDD85852; **Location:** countryCode: IT; stateProvince: Liguria; county: Imperia; municipality: San Biagio della Cima; verbatimElevation: 88 m; verbatimLatitude: 43.8219; verbatimLongitude: 7.6538; **Identification:** identifiedBy: Sirio Gamba; **Event:** eventDate: 2022-03-28; **Record Level:** collectionCode: SGC
- **Type status:** Other material. **Occurrence:** recordedBy: Simone Flaminio; sex: 2 males, 1 female; occurrenceID: FFF779B6-70E9-5EDD-87CE-341A75BFC37C; **Location:** countryCode: IT; stateProvince: Calabria; county: Crotone; municipality: Cutro; verbatimLatitude: 38.9397; verbatimLongitude: 16.9622; **Identification:** identifiedBy: Simone Flaminio; **Event:** eventDate: 2022-04-21; **Record Level:** collectionCode: SFC
- **Type status:** Other material. **Occurrence:** recordedBy: Simone Flaminio; sex: 3 males; occurrenceID: 64C742CF-EED6-5E56-8E29-30752A8E092E; **Location:** countryCode: IT; stateProvince: Calabria; county: Crotone; municipality: Crotone; verbatimLatitude: 39.0105; verbatimLongitude: 17.1783; **Identification:** identifiedBy: Simone Flaminio; **Event:** eventDate: 2022-04-21; **Record Level:** collectionCode: SFC
- **Type status:** Other material. **Occurrence:** recordedBy: Simone Flaminio; sex: 3 males, 1 female; occurrenceID: 66A5FADC-AE56-5C87-8783-68F0EB20DFEC; **Location:** countryCode: IT; stateProvince: Calabria; county: Reggio Calabria; municipality: Caulonia; verbatimLatitude: 38.338; verbatimLongitude: 16.4605; **Identification:** identifiedBy: Simone Flaminio; **Event:** eventDate: 2022-04-23; **Record Level:** collectionCode: SFC

##### Notes

In Italy, *R.sticticum* was formerly reported only from Sicilia and Sardegna (Kasparek 2019, Comba 2019); our records extend its range to continental Italy (Fig. 1A). It is common in the extreme west of Liguria, where it occurs in warm dry places from the French border east at least to Bordighera.

#### Trachusa integra

(Eversmann, 1852)

B0C9EE81-90A3-5EFE-8F39-79E9FC58BBAF

##### Materials

- **Type status:** Other material. **Occurrence:** recordedBy: Marco Selis; sex: 1 male; occurrenceID: 6E78BEED-53C2-5AA7-991E-B5BCCBF5171D; **Location:** countryCode: IT; stateProvince: Lazio; county: Roma; municipality: Roma; locality: Maccarese; verbatimElevation: 30-65 m; verbatimLatitude: 41.8858; verbatimLongitude: 12.2655; **Identification:** identifiedBy: Marco Selis; **Event:** eventDate: 2022-05-22; **Record Level:** collectionCode: MSC
- **Type status:** Other material. **Occurrence:** recordedBy: Marco Selis; sex: 4 males, 6 females; occurrenceID: ECFBEE05-407C-55AE-AEBD-8B088AF54A19; **Location:** countryCode: IT; stateProvince: Lazio; county: Roma; municipality: Roma; locality: Maccarese; verbatimElevation: 30-65 m; verbatimLatitude: 41.8858; verbatimLongitude: 12.2655; **Identification:** identifiedBy: Marco Selis; **Event:** eventDate: 2022-06-14; **Record Level:** collectionCode: MSC, MIB:ZPL
- **Type status:** Other material. **Occurrence:** recordedBy: Maurizio Mei; sex: 1 male; occurrenceID: 4A188994-9BBF-5247-B22A-903F14732890; **Location:** countryCode: IT; stateProvince: Lazio; county: Roma; municipality: Roma; locality: Valle dell'Insugherata; verbatimElevation: 50 m; verbatimLatitude: 41.9592; verbatimLongitude: 12.4352; **Identification:** identifiedBy: Maurizio Mei; **Event:** eventDate: 2015-06-06; **Record Level:** collectionCode: MEC
- **Type status:** Other material. **Occurrence:** recordedBy: Maurizio Mei; sex: 2 females; occurrenceID: 47F1FC8D-F5B0-5AB9-8835-BB23B7091837; **Location:** countryCode: IT; stateProvince: Lazio; county: Roma; municipality: Roma; locality: Valle dell'Insugherata; verbatimElevation: 50 m; verbatimLatitude: 41.9592; verbatimLongitude: 12.4352; **Identification:** identifiedBy: Maurizio Mei; **Event:** eventDate: 2015-06-11; **Record Level:** collectionCode: MZUR
- **Type status:** Other material. **Occurrence:** recordedBy: Maurizio Mei; sex: 1 male; occurrenceID: 66CD8423-AEFC-5DF7-A99C-253408426500; **Location:** countryCode: IT; stateProvince: Lazio; county: Roma; municipality: Roma; locality: Valle dell'Insugherata; verbatimElevation: 50 m; verbatimLatitude: 41.9592; verbatimLongitude: 12.4352; **Identification:** identifiedBy: Maurizio Mei; **Event:** eventDate: 2015-06-22; **Record Level:** collectionCode: MZUR
- **Type status:** Other material. **Occurrence:** recordedBy: Maurizio Mei; sex: 1 female; occurrenceID: 4D6308FC-FCB0-5E04-AD26-3FBC51AC2B14; **Location:** countryCode: IT; stateProvince: Lazio; county: Roma; municipality: Roma; locality: Parco della Caffarella; verbatimElevation: 33 m; verbatimLatitude: 41.8651; verbatimLongitude: 12.5254; **Identification:** identifiedBy: Maurizio Mei; **Event:** eventDate: 2022-05-25; **Record Level:** collectionCode: MZUR
- **Type status:** Other material. **Occurrence:** recordedBy: Maurizio Mei; sex: 1 female; occurrenceID: 4C244FFE-FDC8-50D1-AFBC-F26DAD05DE92; **Location:** countryCode: IT; stateProvince: Lazio; county: Roma; municipality: Roma; locality: Parco di Tor Tre Teste; verbatimElevation: 50 m; verbatimLatitude: 41.8794; verbatimLongitude: 12.5873; **Identification:** identifiedBy: Maurizio Mei; **Event:** eventDate: 2022-06-05; **Record Level:** collectionCode: MZUR
- **Type status:** Other material. **Occurrence:** recordedBy: Maurizio Mei; sex: 2 males, 2 females; occurrenceID: 12DF3A96-BA1C-5D67-B9E4-D45B8830CE65; **Location:** countryCode: IT; stateProvince: Sardegna; county: Oristano; municipality: San Giovanni di Sinis; verbatimElevation: 10 m; verbatimLatitude: 39.8697; verbatimLongitude: 8.4395; **Identification:** identifiedBy: Maurizio Mei; **Event:** eventDate: 2016-06-24/07-15; **Record Level:** collectionCode: MZUR
- **Type status:** Other material. **Occurrence:** recordedBy: Maurizio Mei; sex: 4 females; occurrenceID: 2AA0E6DF-46F9-55E7-AFA6-9BECEF5EEF45; **Location:** countryCode: IT; stateProvince: Sardegna; county: Oristano; municipality: Oristano; locality: mouth of the river Tirso; verbatimElevation: 5 m; verbatimLatitude: 39.8878; verbatimLongitude: 8.5407; **Identification:** identifiedBy: Maurizio Mei; **Event:** eventDate: 2022-07-08; **Record Level:** collectionCode: MEC, MZUR

##### Notes

*Trachusaintegra* was considered conspecific with *Trachusainterrupta* (Fabricius, 1781) until Kasparek (2020) returned it to valid species status. Its range, as described in Kasparek (2020), is discontinuous and includes parts of southern France, the southern Balkans, inner Anatolia, Crimea and parts of southern Russia. *Trachusaintegra* is here reported for the first time from Italy. Eight specimens, two from Sardegna and six from Lazio, were confirmed by DNA barcoding: the match with reference sequences is between 99.84% and 98.32% (mean 99.05%). Images: Suppl. material 2, Figs. S5 and S6.

#### Ammobates vinctus

Gerstaecker, 1869

5DEBDDD4-0A4C-54B7-9047-D8EA69983514

##### Materials

- **Type status:** Other material. **Occurrence:** recordedBy: Marco Selis; sex: 5 males; occurrenceID: 96F813C0-F98F-55B9-BCD3-CE40664014EE; **Location:** countryCode: IT; stateProvince: Lazio; county: Viterbo; municipality: Viterbo; locality: Necropoli etrusca di Norchia; verbatimElevation: 130 m; verbatimLatitude: 42.3451; verbatimLongitude: 11.9443; **Identification:** identifiedBy: Marco Selis; **Event:** eventDate: 2022-07-22; **Record Level:** collectionCode: MSC
- **Type status:** Other material. **Occurrence:** recordedBy: Marco Selis; sex: 7 males, 2 females; occurrenceID: 46A18553-FABE-5955-A9E3-2CC9884C2828; **Location:** countryCode: IT; stateProvince: Lazio; county: Viterbo; municipality: Viterbo; locality: Necropoli etrusca di Norchia; verbatimElevation: 130 m; verbatimLatitude: 42.3451; verbatimLongitude: 11.9443; **Identification:** identifiedBy: Marco Selis; **Event:** eventDate: 2022-07-27; **Record Level:** collectionCode: MSC

##### Notes

All specimens were collected on *Centaureasolstitialis*, flying together with *Tetraloniagraja* and *T.julliani*. The Italian literature records of *A.vinctus* are all from northern Italy, more precisely from Piemonte and Trentino Alto Adige/Südtirol (Comba 2019 and references therein). Our records appear to be the first from Lazio and central Italy. Images: Suppl. material 2, Figs. S8–S10.

#### Anthophora affinis

Brullé, 1832

115D7CDC-EC39-5BC4-B015-AD04D8E70398

##### Materials

- **Type status:** Other material. **Occurrence:** recordedBy: Sirio Gamba; sex: 1 female; occurrenceID: 2F6A4BED-0898-5E4D-8D6B-0E9664425347; **Location:** countryCode: IT; stateProvince: Liguria; county: Imperia; municipality: Camporosso; verbatimElevation: 366 m; verbatimLatitude: 43.8236; verbatimLongitude: 7.6064; **Identification:** identifiedBy: Sirio Gamba, vid. P. Rasmont; **Event:** eventDate: 2018-05-13; **Record Level:** collectionCode: SGC
- **Type status:** Other material. **Occurrence:** recordedBy: Sirio Gamba; sex: 1 male; occurrenceID: 0D5FA78E-2F74-5670-BD9C-7F155E4240C7; **Location:** countryCode: IT; stateProvince: Liguria; county: Imperia; municipality: Apricale; verbatimElevation: 550 m; verbatimLatitude: 43.9047; verbatimLongitude: 7.6819; **Identification:** identifiedBy: Sirio Gamba, vid. P. Rasmont; **Event:** eventDate: 2018-05-08; **Record Level:** collectionCode: SGC
- **Type status:** Other material. **Occurrence:** recordedBy: Marco Bonifacino; sex: 1 male; occurrenceID: 5605EECE-EEE7-5EA5-8740-6D64DD74275D; **Location:** countryCode: IT; stateProvince: Liguria; county: Imperia; municipality: Pigna; verbatimElevation: 1235 m; verbatimLatitude: 43.9578; verbatimLongitude: 7.6278; **Identification:** identifiedBy: Marco Bonifacino; **Event:** eventDate: 2020-05-18; **Record Level:** collectionCode: MBC

##### Notes

The occurrence of *A.affinis* in Italy was for a long time unclear, mostly due to persistent confusion with other species, chiefly *A.mucida* Gribodo, 1873 and *A.agama* Radoszkowski, 1869. The species is said to be of uncertain presence in Italy by Rasmont and Dehon (2015). Images: Suppl. material 2, Figs. S19–S22.

#### Anthophora calcarata

Lepeletier, 1841

E893C619-B414-50B5-99E2-8E55AB7FC4F6

##### Materials

- **Type status:** Other material. **Occurrence:** recordedBy: Maurizio Mei; sex: 3 males; occurrenceID: 2FCBA83D-9D1B-5F4D-B020-564BCB2F8EF7; **Location:** countryCode: IT; stateProvince: Sicilia; county: Trapani; municipality: Pantelleria; locality: Montagna Grande; verbatimElevation: 770 m; verbatimLatitude: 36.7794; verbatimLongitude: 11.9998; **Identification:** identifiedBy: Maurizio Mei; **Event:** eventDate: 1990-03-31; **Record Level:** collectionCode: MEC, MZUR

##### Notes

We are not aware of any previous solid records of *A.calcarata* from Sicilia. The species is mentioned in a catalogue of Sicilian bees by De Stefani (1895), without details. The absence from this same list of the similar *Anthophoracrassipes* Lepeletier, 1841, which occurs widely in Sicilia, is curious and might hint at a misidentification. However, since De Stefani's collection is thought to be lost, there is no way of verifying the identity of De Stefani's specimens. Our record confirms the occurrence of *A.calcarata*, if not on the mainland of Sicilia, at least on the surrounding islands. Images: Suppl. material 2, Figs. S11 and S12.

#### Anthophora dufourii

Lepeletier, 1841

692A182C-4609-55CE-A7C9-8AA58C6FDF5A

##### Materials

- **Type status:** Other material. **Occurrence:** recordedBy: Marco Bonifacino; sex: 1 male, 1 female; occurrenceID: C0512017-6BFB-5B71-8767-215FBB18893C; **Location:** countryCode: IT; stateProvince: Liguria; county: Savona; municipality: Dego; verbatimElevation: 460 m; verbatimLatitude: 44.4571; verbatimLongitude: 8.3441; **Identification:** identifiedBy: Marco Bonifacino; **Event:** eventDate: 2020-05-06; **Record Level:** collectionCode: MBC

##### Notes

There are very few literature records of *A.dufourii* from Italy, all very old (Comba 2019 and references therein). The species has been found in Liguria in the past: in the Sagemehl collection of the University of Tartu Natural History Museum, there is a female specimen from Sanremo without collection date, but in all evidence collected over 100 years ago (record accessible at https://elurikkus.ee/generic-hub/occurrences/429ad39e-d12c-4b24-af8e-c24e175aad07?lang=en). Images: Suppl. material 2, Figs. S13–S15.

#### Anthophora femorata

(Olivier, 1789)

4CF7C71E-D9EB-5C49-A27A-668C154AE4F0

##### Materials

- **Type status:** Other material. **Occurrence:** recordedBy: Marco Selis; sex: 1 male; occurrenceID: FDEAF205-1DA8-54AD-A69A-689FFCF62220; **Location:** countryCode: IT; stateProvince: Lazio; county: Roma; municipality: Manziana; locality: Bosco Macchia Grande; verbatimElevation: 330-370 m; verbatimLatitude: 42.1233; verbatimLongitude: 12.1147; **Identification:** identifiedBy: Marco Selis; **Event:** eventDate: 2019-06-25; **Record Level:** collectionCode: MSC
- **Type status:** Other material. **Occurrence:** recordedBy: Marco Selis; sex: 2 males, 3 females; occurrenceID: AACD9DCA-E9CC-5989-B0A8-F68A2FA6E045; **Location:** countryCode: IT; stateProvince: Lazio; county: Roma; municipality: Manziana; locality: Bosco Macchia Grande; verbatimElevation: 330-370 m; verbatimLatitude: 42.1233; verbatimLongitude: 12.1147; **Identification:** identifiedBy: Marco Selis; **Event:** eventDate: 2020-06-18; **Record Level:** collectionCode: MSC
- **Type status:** Other material. **Occurrence:** recordedBy: Marco Selis; sex: 1 female; occurrenceID: EB99A0BD-4B9F-5B58-92E2-FB613B2B1516; **Location:** countryCode: IT; stateProvince: Lazio; county: Roma; municipality: Manziana; locality: Bosco Macchia Grande; verbatimElevation: 330-370 m; verbatimLatitude: 42.1233; verbatimLongitude: 12.1147; **Identification:** identifiedBy: Marco Selis; **Event:** eventDate: 2021-06-12; **Record Level:** collectionCode: MSC
- **Type status:** Other material. **Occurrence:** recordedBy: Marco Selis; sex: 1 male; occurrenceID: 5E0C2670-325F-511E-9F0B-A99BE71D109A; **Location:** countryCode: IT; stateProvince: Lazio; county: Roma; municipality: Manziana; locality: Bosco Macchia Grande; verbatimElevation: 330-370 m; verbatimLatitude: 42.1233; verbatimLongitude: 12.1147; **Identification:** identifiedBy: Marco Selis; **Event:** eventDate: 2021-06-22; **Record Level:** collectionCode: MSC
- **Type status:** Other material. **Occurrence:** recordedBy: Marco Selis; sex: 1 female; occurrenceID: 3528D9D3-A6A0-526A-83A9-FD119E08C380; **Location:** countryCode: IT; stateProvince: Lazio; county: Roma; municipality: Manziana; locality: Bosco Macchia Grande; verbatimElevation: 330-370 m; verbatimLatitude: 42.1233; verbatimLongitude: 12.1147; **Identification:** identifiedBy: Marco Selis; **Event:** eventDate: 2022-06-01; **Record Level:** collectionCode: MSC
- **Type status:** Other material. **Occurrence:** recordedBy: Marco Selis; sex: 2 males, 1 female; occurrenceID: CE619FBC-D2DD-5F22-ABB0-798E7C334F2B; **Location:** countryCode: IT; stateProvince: Lazio; county: Viterbo; municipality: Viterbo; locality: Valle dell'Arcionello; verbatimElevation: 460 m; verbatimLatitude: 42.4181; verbatimLongitude: 12.1419; **Identification:** identifiedBy: Marco Selis; **Event:** eventDate: 2021-06-21; **Record Level:** collectionCode: MSC
- **Type status:** Other material. **Occurrence:** recordedBy: Aleida Ascenzi, Pierfilippo Cerretti; sex: 1 male; occurrenceID: 4B7C8C0B-7221-5EE5-BBCE-B26B881355AD; **Location:** countryCode: IT; stateProvince: Lazio; county: Roma; municipality: Roma; locality: Castel Porziano, Grotta Romagnola; verbatimElevation: 35 m; verbatimLatitude: 41.7541; verbatimLongitude: 12.4305; **Identification:** identifiedBy: Maurizio Mei; **Event:** eventDate: 2022-05-6/20; **Record Level:** collectionCode: MZUR

##### Notes

The literature records of *A.femorata* from Italy (Comba 2019) are few, all very old and somewhat questionable. Our records are the only recent ones known to us.

#### Anthophora fulvitarsis

Brullé, 1832

B2F2EAD4-191B-58E0-8B72-D53A662B2541

##### Materials

- **Type status:** Other material. **Occurrence:** recordedBy: Sirio Gamba; sex: 1 female; occurrenceID: 20DC669A-B955-5E66-ADFA-8F9F14EF2EE6; **Location:** countryCode: IT; stateProvince: Liguria; county: Imperia; municipality: Ventimiglia; verbatimElevation: 6 m; verbatimLatitude: 43.7878; verbatimLongitude: 7.6289; **Identification:** identifiedBy: Sirio Gamba; **Event:** eventDate: 2022-05-10; **Record Level:** collectionCode: SGC
- **Type status:** Other material. **Occurrence:** recordedBy: Sirio Gamba; sex: 1 male; occurrenceID: 85EA0B68-68E7-5B92-A00A-1A69E5EAF2C3; **Location:** countryCode: IT; stateProvince: Liguria; county: Imperia; municipality: Ventimiglia; verbatimElevation: 6 m; verbatimLatitude: 43.7878; verbatimLongitude: 7.6289; **Identification:** identifiedBy: Sirio Gamba; **Event:** eventDate: 2022-05-11; **Record Level:** collectionCode: SGC
- **Type status:** Other material. **Occurrence:** recordedBy: Sirio Gamba; sex: 1 male; occurrenceID: 9BFF57A4-887D-5A2F-BEF8-BC1BF938F64F; **Location:** countryCode: IT; stateProvince: Liguria; county: Imperia; municipality: Ventimiglia; verbatimElevation: 6 m; verbatimLatitude: 43.7878; verbatimLongitude: 7.6289; **Identification:** identifiedBy: Sirio Gamba; **Event:** eventDate: 2022-05-12; **Record Level:** collectionCode: SGC

##### Notes

Our specimens were found in an urban setting, in an abandoned railway yard. We are not aware of any other recent record of *A.fulvitarsis* in Italy. According to Comba (2019), the only literature record, from Sicilia, is in Sichel (1860). Images: Suppl. material 2, Figs. S16–S18.

#### Bombus hypnorum

(Linnaeus, 1758)

350F7365-DFDC-585E-8D3C-E701D929AF6E

##### Materials

- **Type status:** Other material. **Occurrence:** recordedBy: Paolo Biella; sex: 1 male; occurrenceID: C1990E9B-2990-552D-BF31-0BC1919846B2; **Location:** countryCode: IT; stateProvince: Abruzzo; county: Teramo; municipality: Isola del Gran Sasso; verbatimElevation: 2095 m; verbatimLatitude: 42.4511; verbatimLongitude: 13.6223; **Identification:** identifiedBy: Paolo Biella; **Event:** eventDate: 2020-06-30
- **Type status:** Other material. **Occurrence:** recordedBy: Marco Bonifacino; sex: 1 female; occurrenceID: 78C5BD47-B478-5F73-94D6-A9D566AA14CC; **Location:** countryCode: IT; stateProvince: Abruzzo; county: L'Aquila; municipality: Campotosto; verbatimElevation: 1730 m; verbatimLatitude: 42.5729; verbatimLongitude: 13.39; **Identification:** identifiedBy: Marco Bonifacino; **Event:** eventDate: 2021-06-15; **Record Level:** collectionCode: MBC
- **Type status:** Other material. **Occurrence:** recordedBy: Marco Bonifacino; sex: 1 male; occurrenceID: 7A77B5CC-DDB7-58B3-A5A4-C9FD8BB12E4F; **Location:** countryCode: IT; stateProvince: Abruzzo; county: L'Aquila; municipality: Barrea; verbatimElevation: 1160 m; verbatimLatitude: 41.7456; verbatimLongitude: 13.9691; **Identification:** identifiedBy: Marco Bonifacino; **Event:** eventDate: 2022-06-13; **Record Level:** collectionCode: MBC
- **Type status:** Other material. **Occurrence:** recordedBy: Maurizio Mei; sex: 1 male; occurrenceID: 4791DC97-7192-5743-A078-3487CBA4A6DF; **Location:** countryCode: IT; stateProvince: Abruzzo; county: L'Aquila; municipality: Cappadocia; locality: road to Fonte Maiura; verbatimElevation: 1130 m; verbatimLatitude: 41.9991; verbatimLongitude: 13.2854; **Identification:** identifiedBy: Maurizio Mei; **Event:** eventDate: 2021-08-02; **Record Level:** collectionCode: MEC
- **Type status:** Other material. **Occurrence:** recordedBy: Maurizio Mei; sex: 1 male; occurrenceID: 259C8371-AAC5-59A0-A0E1-CFEA32E360CF; **Location:** countryCode: IT; stateProvince: Abruzzo; county: L'Aquila; municipality: Cappadocia; locality: Camporotondo, La Ceria; verbatimElevation: 1540 m; verbatimLatitude: 41.9713; verbatimLongitude: 13.29; **Identification:** identifiedBy: Maurizio Mei; **Event:** eventDate: 2021-08-06; **Record Level:** collectionCode: MEC
- **Type status:** Other material. **Occurrence:** recordedBy: Pierfilippo Cerretti; sex: 1 female; occurrenceID: 4E6A1CEC-C852-5F3A-B92E-007D09EE16F5; **Location:** countryCode: IT; stateProvince: Abruzzo; county: Teramo; municipality: Fano Adriano; locality: Incodaro; verbatimElevation: 1400 m; verbatimLatitude: 42.5123; verbatimLongitude: 13.4735; **Identification:** identifiedBy: Maurizio Mei; **Event:** eventDate: 2022-06-23; **Record Level:** collectionCode: MZUR

##### Notes

The specimens from Campotosto, Barrea, Camporotondo and Fano Adriano were collected in grassy clearings at the edge of beech forest. The male from Cappadocia was collected on *Cirsium* sp., along a road running through orchards and open mixed woodland. Prior to our records, *B.hypnorum* was known to occur throughout the Italian Alps and adjacent foothills, in the Northern Apennines in the Provinces of Genova (verified records in GBIF), Pavia, Piacenza (M. Cornalba personal observations), Massa Carrara (Intoppa et al. 1995), Lucca (P. Biella, personal observations) and Forlì-Cesena (Quaranta et al. 2004), perhaps in the Provinces of Pisa and Firenze (Rossi 1790) and, furthermore, in scattered localities in the plains of Lombardia, Veneto and Friuli Venezia Giulia (Pensa 1832, Intoppa et al. 1995, Barbattini et al. 2006) M. Cornalba personal observations, verified records in GBIF and iNaturalist). The occurrence of the species throughout Abruzzo, over 200 km from the nearest Italian record, constitutes a significant extension of the range of *B.hypnorum* and raises several questions, all related to each other. Is *B.hypnorum* a recent arrival in Abruzzo or was it just overlooked before? Does the species occur elsewhere in the Apennines, particularly between the Northern Apennines and Abruzzo? The ecological conditions prevailing throughout this portion of the mountain chain would seem conducive to the presence of *B.hypnorum*. Finally, what is the origin of the Abruzzo population?

#### Epeoloides coecutiens

(Fabricius, 1775)

A75F12AB-8DC7-5230-AD15-35296E058D94

##### Materials

- **Type status:** Other material. **Occurrence:** recordedBy: Maurizio Mei; sex: 1 female; occurrenceID: DDA8DCEF-0412-5D83-82CE-37D325F26737; **Location:** countryCode: IT; stateProvince: Lazio; county: Roma; municipality: Anticoli Corrado; verbatimElevation: 320 m; verbatimLatitude: 42.0185; verbatimLongitude: 12.9929; **Identification:** identifiedBy: Maurizio Mei; **Event:** eventDate: 2021-07-16; **Record Level:** collectionCode: MZUR

##### Notes

On flowers of *Lysimachiavulgaris*, in a clearing along the banks of the River Aniene, flying together with *Macropiseuropaea*. Our records significantly extend the range of the species, which had previously been recorded in Italy – very sporadically – only in the north (Comba 2019 and references therein).

#### Epeolus productulus

Bischoff, 1930

66378A1F-FD0C-5A8D-9B49-3D44E5888E97

##### Materials

- **Type status:** Other material. **Occurrence:** recordedBy: Marco Selis; sex: 7 males, 12 females; occurrenceID: 9283AF56-A9B6-5570-8A8A-7E98D2E81481; **Location:** countryCode: IT; stateProvince: Lazio; county: Roma; municipality: Manziana; locality: Bosco Macchia Grande; verbatimElevation: 330-370 m; verbatimLatitude: 42.1233; verbatimLongitude: 12.1147; **Identification:** identifiedBy: Marco Selis, vid. R. Le Divelec; **Event:** eventDate: 2022-06-01; **Record Level:** collectionCode: MSC

##### Notes

Found resting on grass and flowers at sunset, together with *Colletesmlokossewiczi*. Prior to our find, *E.productulus* was documented in Italy from Valle d’Aosta (Amiet et al. 2007), Piemonte (Schwarz et al. 1999, Bogusch and Hadrava 2018) and Emilia-Romagna (Bogusch and Hadrava 2018). We sequenced one male and one female and the resulting sequences seem to be the first for *E.productulus*. The nearest species for which sequences are deposited in BOLD is *Epeolusvariegatus* (average match 96.89%, range 97.99%-92.56%).

#### Eucera furfurea

Vachal, 1907

2A6AF792-4293-575D-9532-D8868934211E

##### Materials

- **Type status:** Other material. **Occurrence:** recordedBy: Maurizio Bollino; sex: 19 males; occurrenceID: 849D1FE9-C120-5592-B64B-63CE6B402176; **Location:** countryCode: IT; stateProvince: Puglia; county: Lecce; municipality: Vernole; locality: Termetito; verbatimElevation: 5 m; verbatimLatitude: 40.3391; verbatimLongitude: 18.365; **Identification:** identifiedBy: Marco Selis, vid. A. Dorchin; **Event:** eventDate: 2021-05-08/10; **Record Level:** collectionCode: MSC
- **Type status:** Other material. **Occurrence:** recordedBy: Maurizio Bollino; sex: 2 males; occurrenceID: 308C0542-CE01-5D1B-9635-233F1B278AF6; **Location:** countryCode: IT; stateProvince: Puglia; county: Lecce; municipality: Vernole; locality: Termetito; verbatimElevation: 5 m; verbatimLatitude: 40.3391; verbatimLongitude: 18.365; **Identification:** identifiedBy: Marco Selis, vid. A. Dorchin; **Event:** eventDate: 2021-05-12/13; **Record Level:** collectionCode: MSC
- **Type status:** Other material. **Occurrence:** recordedBy: Maurizio Bollino; sex: 1 male; occurrenceID: E39E8B2F-ED8B-5E2C-A04A-4863A064E138; **Location:** countryCode: IT; stateProvince: Puglia; county: Lecce; municipality: Vernole; locality: Termetito; verbatimElevation: 5 m; verbatimLatitude: 40.3391; verbatimLongitude: 18.365; **Identification:** identifiedBy: Marco Selis, vid. A. Dorchin; **Event:** eventDate: 2021-05-16/17; **Record Level:** collectionCode: MSC

##### Notes

We are not aware of any published Italian records of *E.furfurea*. The species has been recorded from Puglia and Sicilia (S. Risch, personal communication 2022).

#### Eucera pannonica

Mocsáry, 1878

732FC356-6CC1-5686-BE62-B43C27385B46

##### Materials

- **Type status:** Other material. **Occurrence:** recordedBy: Maurizio Mei; sex: 1 female; occurrenceID: 686CED32-F98D-581D-8580-EE73F2B9C71A; **Location:** countryCode: IT; stateProvince: Lazio; county: Roma; municipality: Tivoli; locality: Colle Vescovo; verbatimElevation: 408 m; verbatimLatitude: 41.9631; verbatimLongitude: 12.8126; **Identification:** identifiedBy: Maurizio Mei; **Event:** eventDate: 2021-06-20; **Record Level:** collectionCode: MZUR
- **Type status:** Other material. **Occurrence:** recordedBy: Maurizio Mei; sex: 1 female; occurrenceID: B6AE05A6-A7DF-5CC2-B012-57CA09A6AEA9; **Location:** countryCode: IT; stateProvince: Lazio; county: Roma; municipality: Roma; locality: Parco della Caffarella; verbatimElevation: 33 m; verbatimLatitude: 41.8651; verbatimLongitude: 12.5254; **Identification:** identifiedBy: Maurizio Mei; **Event:** eventDate: 2022-05-25; **Record Level:** collectionCode: MZUR
- **Type status:** Other material. **Occurrence:** recordedBy: Aleida Ascenzi, Pierfilippo Cerretti; sex: 12 males; occurrenceID: 25C0783E-F374-529A-9894-22AB0C5231C8; **Location:** countryCode: IT; stateProvince: Lazio; county: Roma; municipality: Roma; locality: Castel Porziano, Grotta Romagnola; verbatimElevation: 35 m; verbatimLatitude: 41.7541; verbatimLongitude: 12.4305; **Identification:** identifiedBy: Maurizio Mei; **Event:** eventDate: 2022-05-6/20; **Record Level:** collectionCode: MZUR
- **Type status:** Other material. **Occurrence:** recordedBy: Marco Selis; sex: 1 male; occurrenceID: DD41BB6E-D738-5B93-9200-AE978BF5B52D; **Location:** countryCode: IT; stateProvince: Lazio; county: Viterbo; municipality: Viterbo; locality: Necropoli etrusca di Norchia; verbatimElevation: 130-160 m; verbatimLatitude: 42.3381; verbatimLongitude: 11.9456; **Identification:** identifiedBy: Marco Selis, vid. A. Dorchin; **Event:** eventDate: 2020-05-08/10; **Record Level:** collectionCode: MSC
- **Type status:** Other material. **Occurrence:** recordedBy: Marco Selis; sex: 4 males; occurrenceID: 4D0A87F9-5DC4-56B6-A9E2-4E35F1E7B4C7; **Location:** countryCode: IT; stateProvince: Lazio; county: Viterbo; municipality: Viterbo; locality: Necropoli etrusca di Norchia; verbatimElevation: 130-160 m; verbatimLatitude: 42.3381; verbatimLongitude: 11.9456; **Identification:** identifiedBy: Marco Selis; **Event:** eventDate: 2022-05-12; **Record Level:** collectionCode: MSC
- **Type status:** Other material. **Occurrence:** recordedBy: Marco Selis; sex: 8 males, 2 females; occurrenceID: E876605D-89F8-5BDD-933D-2DBB214C168B; **Location:** countryCode: IT; stateProvince: Lazio; county: Roma; municipality: Santa Severa; locality: Rio Fiume; verbatimElevation: 40 m; verbatimLatitude: 42.0617; verbatimLongitude: 11.9524; **Identification:** identifiedBy: Marco Selis; **Event:** eventDate: 2022-05-15; eventRemarks: on *Silybummarianum*; **Record Level:** collectionCode: MSC
- **Type status:** Other material. **Occurrence:** recordedBy: Marco Bonifacino; sex: 2 males, 2 females; occurrenceID: DAAE607A-40BD-530C-9766-9679B83C2E5D; **Location:** countryCode: IT; stateProvince: Abruzzo; county: L'Aquila; municipality: Pescina; verbatimElevation: 820 m; verbatimLatitude: 42.0352; verbatimLongitude: 13.6747; **Identification:** identifiedBy: Marco Bonifacino; **Event:** eventDate: 2022-05-28; eventRemarks: on *Carduus* sp.; **Record Level:** collectionCode: MBC

##### Notes

*E.pannonica* has been recorded from Mount Gargano in Puglia and from Sicilia (S. Risch, personal communication 2022). Our records seem to be the first from Lazio and Abruzzo.

#### Eucera terminata

Pérez, 1895

19056F84-8685-544B-8517-A766D2C15416

##### Materials

- **Type status:** Other material. **Occurrence:** recordedBy: Marco Selis; sex: 1 male; occurrenceID: 7DD2D243-4D8F-5093-ACAD-5346DC8A59DF; **Location:** countryCode: IT; stateProvince: Lazio; county: Viterbo; municipality: Viterbo; locality: Necropoli etrusca di Norchia; verbatimElevation: 130 m; verbatimLatitude: 42.3381; verbatimLongitude: 11.9456; **Identification:** identifiedBy: Achik Dorchin; **Event:** eventDate: 2020-05-08/10; **Record Level:** collectionCode: MSC
- **Type status:** Other material. **Occurrence:** recordedBy: Marco Selis; sex: 6 males; occurrenceID: 851CFA57-F6C0-58A0-A1F2-34270367F8DC; **Location:** countryCode: IT; stateProvince: Lazio; county: Viterbo; municipality: Viterbo; locality: Necropoli etrusca di Norchia; verbatimElevation: 130 m; verbatimLatitude: 42.3381; verbatimLongitude: 11.9456; **Identification:** identifiedBy: Marco Selis; **Event:** eventDate: 2022-05-04; **Record Level:** collectionCode: MSC
- **Type status:** Other material. **Occurrence:** recordedBy: Marco Selis; sex: 1 male; occurrenceID: 7BB9D112-3F26-53D5-BE8F-D40C14803BAA; **Location:** countryCode: IT; stateProvince: Lazio; county: Viterbo; municipality: Viterbo; locality: Necropoli etrusca di Norchia; verbatimElevation: 130 m; verbatimLatitude: 42.3381; verbatimLongitude: 11.9456; **Identification:** identifiedBy: Marco Selis; **Event:** eventDate: 2022-05-12; **Record Level:** collectionCode: MSC
- **Type status:** Other material. **Occurrence:** recordedBy: Maurizio Mei; sex: 1 male; occurrenceID: 15963F8E-DB4E-5F7E-82D5-A31DFAB8D761; **Location:** countryCode: IT; stateProvince: Lazio; county: Roma; municipality: Roma; locality: Tenuta della Cervelletta; verbatimElevation: 24 m; verbatimLatitude: 41.9144; verbatimLongitude: 12.5852; **Identification:** identifiedBy: Maurizio Mei; **Event:** eventDate: 2009-05-11; **Record Level:** collectionCode: MZUR
- **Type status:** Other material. **Occurrence:** recordedBy: Maurizio Mei; sex: 2 males; occurrenceID: 4A76BE1C-7D45-58B5-9F09-3977C29039C3; **Location:** countryCode: IT; stateProvince: Lazio; county: Roma; municipality: Roma; locality: Tenuta della Cervelletta; verbatimElevation: 24 m; verbatimLatitude: 41.9144; verbatimLongitude: 12.5852; **Identification:** identifiedBy: Maurizio Mei; **Event:** eventDate: 2014-04-25; **Record Level:** collectionCode: MEC, MZUR
- **Type status:** Other material. **Occurrence:** recordedBy: Maurizio Mei; sex: 1 male; occurrenceID: E558C19D-F6A6-5C52-8230-A591DAFEB5B2; **Location:** countryCode: IT; stateProvince: Lazio; county: Roma; municipality: Roma; locality: Tor Sapienza; verbatimElevation: 35 m; verbatimLatitude: 41.9021; verbatimLongitude: 12.5826; **Identification:** identifiedBy: Maurizio Mei; **Event:** eventDate: 2022-04-04; **Record Level:** collectionCode: MZUR
- **Type status:** Other material. **Occurrence:** recordedBy: Maurizio Mei; sex: 3 males; occurrenceID: F8DF6BE2-7F52-5ED5-B98E-85B71ABB5222; **Location:** countryCode: IT; stateProvince: Lazio; county: Roma; municipality: Roma; locality: Parco degli Acquedotti; verbatimElevation: 60 m; verbatimLatitude: 41.8435; verbatimLongitude: 12.567; **Identification:** identifiedBy: Maurizio Mei; **Event:** eventDate: 2022-05-09; **Record Level:** collectionCode: MEC, MZUR
- **Type status:** Other material. **Occurrence:** recordedBy: Maurizio Mei; sex: 1 male; occurrenceID: B26314B8-78DB-5B4C-817A-6B29FCD76EDE; **Location:** countryCode: IT; stateProvince: Lazio; county: Roma; municipality: San Gregorio da Sassola; verbatimElevation: 596 m; verbatimLatitude: 41.9319; verbatimLongitude: 12.8665; **Identification:** identifiedBy: Maurizio Mei; **Event:** eventDate: 2013-04-29; **Record Level:** collectionCode: MZUR
- **Type status:** Other material. **Occurrence:** recordedBy: Federico Hartig; sex: 1 male; occurrenceID: 31DF5353-0C42-51E1-AE98-A135EC2E246F; **Location:** countryCode: IT; stateProvince: Sicilia; county: Messina; municipality: Taormina; locality: Monte Ziretto; verbatimElevation: 200 m; **Identification:** identifiedBy: Maurizio Mei; **Event:** eventDate: 1950-03-26; **Record Level:** collectionCode: MZUR
- **Type status:** Other material. **Occurrence:** recordedBy: Omero Castellani; sex: 1 male; occurrenceID: 11AAC788-674E-5EDF-8562-F78B2E205886; **Location:** countryCode: IT; stateProvince: Lazio; county: Frosinone; municipality: Fiuggi; locality: Lago di Canterno; verbatimElevation: 540 m; **Identification:** identifiedBy: Maurizio Mei; **Event:** eventDate: 1938-05-01; **Record Level:** collectionCode: MZUR

##### Notes

This species has generally been known under the name *Euceraobsoleta* Pérez, 1911 which, however, has been synonymised with *E.terminata* by Dorchin (2023). In Italy, *E.terminata* has often been confused with various species of the subgenus Pteneucera sensu Tkalců (1984). The type of *E.terminata* is from Sicilia and the species has been recorded also elsewhere in central and southern Italy (S. Risch, personal communication 2022). Since the latter records do not seem to have been published, we thought it might be useful to publish ours.

#### Nomada duplex

Smith, 1854

E9F6CBB5-BFEC-53B0-B62D-607215C74135

##### Materials

- **Type status:** Other material. **Occurrence:** recordedBy: A. Giacò; sex: 1 male; occurrenceID: 900D7105-36D6-50A4-9BBF-140A5D67A30E; **Location:** countryCode: IT; stateProvince: Toscana; county: Pisa; municipality: Pisa; verbatimLatitude: 43.708; verbatimLongitude: 10.4752; **Identification:** identifiedBy: Simone Flaminio; **Event:** eventDate: 2022-03-13; **Record Level:** collectionCode: SFC
- **Type status:** Other material. **Occurrence:** recordedBy: A. Femia; sex: 1 female; occurrenceID: D0C9438C-C131-516F-AD81-6E5056D9A5B2; **Location:** countryCode: IT; stateProvince: Toscana; county: Firenze; municipality: Sesto Fiorentino; verbatimElevation: 35 m; verbatimLatitude: 43.8144; verbatimLongitude: 11.198; **Identification:** identifiedBy: Marco Selis; **Event:** eventDate: 2021-03-27; **Record Level:** collectionCode: MSC
- **Type status:** Other material. **Occurrence:** recordedBy: A. Marata; sex: 1 female; occurrenceID: A243D406-036A-5455-8FF0-1E85E058A3AA; **Location:** countryCode: IT; stateProvince: Toscana; county: Prato; municipality: Vernio; locality: Luicciana; verbatimElevation: 300 m; verbatimLatitude: 44.0276; verbatimLongitude: 11.1056; **Identification:** identifiedBy: Marco Selis; **Event:** eventDate: 2016-07-22; **Record Level:** collectionCode: MSC
- **Type status:** Other material. **Occurrence:** recordedBy: Simone Flaminio; sex: 1 female; occurrenceID: C5023A20-033B-5E26-83C4-C1433DB5AF67; **Location:** countryCode: IT; stateProvince: Emilia-Romagna; county: Bologna; municipality: Bologna; verbatimLatitude: 44.4506; verbatimLongitude: 11.3675; **Identification:** identifiedBy: Simone Flaminio; **Event:** eventDate: 2022-03-21; **Record Level:** collectionCode: SFC
- **Type status:** Other material. **Occurrence:** recordedBy: G. Pezzi; sex: 2 males; occurrenceID: E781483B-5727-5145-B3DF-9CB033C21259; **Location:** countryCode: IT; stateProvince: Emilia-Romagna; county: Ravenna; municipality: Mezzano; verbatimLatitude: 44.4675; verbatimLongitude: 12.0867; **Identification:** identifiedBy: Marco Selis; **Event:** eventDate: 2019-04-03; **Record Level:** collectionCode: MSC
- **Type status:** Other material. **Occurrence:** recordedBy: E. Pulvirenti; sex: 1 female; occurrenceID: 6D5AA520-BA94-5FEB-AE07-667BFE806AB5; **Location:** countryCode: IT; stateProvince: Lazio; county: Roma; municipality: Guidonia Montecelio; locality: via della Selciatella; verbatimLatitude: 41.9859; verbatimLongitude: 12.7017; **Identification:** identifiedBy: Marco Selis; **Event:** eventDate: 2019-04-06; **Record Level:** collectionCode: MSC
- **Type status:** Other material. **Occurrence:** recordedBy: Maurizio Mei; sex: 1 female; occurrenceID: 321CD2FD-66AE-5108-8A42-D8A72629BCA7; **Location:** countryCode: IT; stateProvince: Lazio; county: Roma; municipality: Roma; locality: Tor Sapienza; verbatimLatitude: 41.9022; verbatimLongitude: 12.5827; **Identification:** identifiedBy: Maurizio Mei; **Event:** eventDate: 2020-04-27; **Record Level:** collectionCode: MZUR

##### Notes

Our records of *N.duplex* appear to be the first for Emilia-Romagna, Toscana and Lazio. In Italy, *N.duplex* has been recorded also from Sicilia, Sardegna, Abruzzo and Marche (Aubert et al. 2010).

#### Nomada flavopicta

(Kirby, 1802)

03389D49-7919-5C56-B32C-12A7DA050558

##### Materials

- **Type status:** Other material. **Occurrence:** recordedBy: Roberto Catania; sex: 1 female; occurrenceID: 63DE8D2F-31AB-5391-A04C-10141BA382A3; **Location:** countryCode: IT; stateProvince: Sicilia; county: Catania; municipality: Randazzo; locality: Lago Gurrida; verbatimElevation: 850 m; verbatimLatitude: 37.8539; verbatimLongitude: 14.8997; **Identification:** identifiedBy: Roberto Catania; **Event:** eventDate: 2020-08-25; **Record Level:** collectionCode: RCC

##### Notes

Our record from the NW slope of Mount Etna appears to be the first for Sicilia. Two of the hosts of *N.flavopicta*, namely *Melittaleporina* (Panzer, 1799) and *M.tricincta* Kirby, 1802, occur on Mount Etna (Nobile and Tomarchio 1997).

#### Nomada hungarica

Dalla Torre & Friese, 1894

FA68ABBC-C7FF-5A1E-B941-B3C5E41B45DA

##### Materials

- **Type status:** Other material. **Occurrence:** recordedBy: Marco Bonifacino; sex: 2 females; occurrenceID: 7BB96CA4-0103-541B-9B87-C22E7DBE5C14; **Location:** countryCode: IT; stateProvince: Liguria; county: Imperia; municipality: Pietrabruna; locality: Boscomare; verbatimElevation: 660 m; verbatimLatitude: 43.8781; verbatimLongitude: 7.8845; **Identification:** identifiedBy: Marco Bonifacino; **Event:** eventDate: 2020-05-22; **Record Level:** collectionCode: MBC
- **Type status:** Other material. **Occurrence:** recordedBy: Marco Bonifacino; sex: 1 female; occurrenceID: 7A5E1441-21C9-5EA9-A51F-CE2713ADFBA8; **Location:** countryCode: IT; stateProvince: Liguria; county: Savona; municipality: Bergeggi; locality: Monte Mao; verbatimElevation: 270 m; verbatimLatitude: 44.2445; verbatimLongitude: 8.4273; **Identification:** identifiedBy: Marco Bonifacino; **Event:** eventDate: 2020-05-25; **Record Level:** collectionCode: MBC
- **Type status:** Other material. **Occurrence:** recordedBy: Marco Selis; sex: 1 male; occurrenceID: 079DAAD9-DA77-5549-8ACE-9ECB14296420; **Location:** countryCode: IT; stateProvince: Lazio; county: Viterbo; municipality: Viterbo; locality: Necropoli etrusca di Norchia; verbatimElevation: 130 m; verbatimLatitude: 42.3381; verbatimLongitude: 11.9456; **Identification:** identifiedBy: Marco Selis; **Event:** eventDate: 2022-05-04; **Record Level:** collectionCode: MSC
- **Type status:** Other material. **Occurrence:** recordedBy: Marco Bonifacino; sex: 1 male; occurrenceID: DE2E2078-B18D-51B3-A3E7-05DBFB2D6E63; **Location:** countryCode: IT; stateProvince: Abruzzo; county: L'Aquila; municipality: Barisciano; verbatimElevation: 865 m; verbatimLatitude: 42.3119; verbatimLongitude: 13.6104; **Identification:** identifiedBy: Marco Bonifacino; **Event:** eventDate: 2021-05-22; **Record Level:** collectionCode: MBC

##### Notes

Ours seem to be the first records of *N.hungarica* from Liguria, Lazio and Abruzzo. The only Italian literature records of *N.hungarica* known to us are from Piemonte (Pagliano 1993) and Sicilia (Nobile 1990, as *Nomadalagrecai* Nobile).

#### Tetralonia inulae

Tkalců, 1979

967B9EDE-2E2C-57CE-A49C-54C689D2EEEA

##### Materials

- **Type status:** Other material. **Occurrence:** recordedBy: Maurizio Cornalba; sex: 1 male; occurrenceID: 18BFBDD3-F511-5330-909D-A6F8F4E931F8; **Location:** countryCode: IT; stateProvince: Lombardia; county: Pavia; municipality: Cecima; verbatimElevation: 688 m; verbatimLatitude: 44.815; verbatimLongitude: 9.0791; **Identification:** identifiedBy: Maurizio Cornalba; **Event:** eventDate: 2018-06-21; eventRemarks: on *Pentanemamontanum*; **Record Level:** collectionCode: MCC
- **Type status:** Other material. **Occurrence:** recordedBy: Maurizio Cornalba; sex: 1 male; occurrenceID: A90BB22B-D13F-50E4-B790-CC60D6ACEBCD; **Location:** countryCode: IT; stateProvince: Lombardia; county: Pavia; municipality: Cecima; verbatimElevation: 688 m; verbatimLatitude: 44.815; verbatimLongitude: 9.0791; **Identification:** identifiedBy: Maurizio Cornalba; **Event:** eventDate: 2018-06-27; eventRemarks: on *Pentanemamontanum*; **Record Level:** collectionCode: MCC
- **Type status:** Other material. **Occurrence:** recordedBy: Maurizio Cornalba; sex: 2 females; occurrenceID: E884B698-B18C-59D3-9073-7849B42300F5; **Location:** countryCode: IT; stateProvince: Lombardia; county: Pavia; municipality: Cecima; verbatimElevation: 650 m; verbatimLatitude: 44.8236; verbatimLongitude: 9.0799; **Identification:** identifiedBy: Maurizio Cornalba; **Event:** eventDate: 2020-07-01; eventRemarks: on *Pentanemaspiraeifolium*; **Record Level:** collectionCode: MCC
- **Type status:** Other material. **Occurrence:** recordedBy: Maurizio Cornalba; sex: 1 female; occurrenceID: 1C024E32-8B16-5895-9C1C-A49720467BEB; **Location:** countryCode: IT; stateProvince: Lombardia; county: Pavia; municipality: Cecima; verbatimElevation: 647 m; verbatimLatitude: 44.8231; verbatimLongitude: 9.0803; **Identification:** identifiedBy: Maurizio Cornalba; **Event:** eventDate: 2022-06-20; eventRemarks: on *Pentanemaspiraeifolium*; **Record Level:** collectionCode: MCC
- **Type status:** Other material. **Occurrence:** recordedBy: Simone Flaminio; sex: 2 males, 2 females; occurrenceID: E75004E1-686D-52A0-BB6F-AF1C967FD006; **Location:** countryCode: IT; stateProvince: Emilia-Romagna; county: Bologna; municipality: Bologna; verbatimElevation: 220 m; verbatimLatitude: 44.4469; verbatimLongitude: 11.3753; **Identification:** identifiedBy: Simone Flaminio; **Event:** eventDate: 2022-06-26; **Record Level:** collectionCode: SFC

##### Notes

At the Cecima site, *T.inulae* co-occurs with *Tetraloniafulvescens* Giraud, 1863 and *Tetraloniaalticincta* (Lepeletier, 1841), the latter flying on average about one month later than *T.inulae*. We obtained a barcode sequence from one of the specimens of *T.inulae* from the Cecima site. Only two short (< 400 bp) sequences labelled *Eucerainulae*, both from Canton Ticino in Switzerland, were available in the BOLD data bank for comparison. They turned out to match our sequence at 99.74%. Aside from them, the nearest match for our sequence, at 97.36%, is a GenBank COI sequence (voucher ad98, accession MG251111) pertaining to a specimen from Erzurum, Turkey, determined as *Euceraalticincta* (Dorchin et al. 2018). Interestingly, the barcode sequences of two *T.alticincta* specimens from the Cecima site turned out to be almost identical to the Turkish one mentioned above (match 99.65%), but distant from the few *T.alticincta* sequences from western Europe available in the BOLD data bank, with matches below 97.5% (Suppl. material 1, Table S1).

## Analysis

Overall, we report records of 368 specimens of 48 bee species, belonging to six families and 23 genera, coming from 14 of the 20 administrative regions of Italy. Most represent first records for Italy or for some of its regions (Table 1). Overall, eight of the species are previously unrecorded in Italy, an additional six previously unrecorded in continental Italy, one in Sicilia and one in Sardegna. A further thirty species are newly reported from at least one administrative region of Italy and we document the continuing occurrence in Italy of five species which had long gone unrecorded in the country (see notes in the “Annotated list of significant records” section). We retrieved long sequences of the COI Folmer region for all the specimens subjected to DNA analyses (see Suppl. material 1). The identification by DNA barcoding was particularly successful, as the resulting sequences yielded high similarity scores to existing sequences of trustworthy species identity (see the notes in the “Annotated list of significant records” section).

## Discussion

In this study, we report eight species of bees which are new for the Italian fauna and, in addition to these, eight which are new for the fauna of mainland Italy, Sardegna or Sicilia. Our results are an important indication of a faunistic richness still awaiting discovery, especially considering that they were obtained by a small group of people, mostly non-specialists, in just a few months. Our results confirm the effectiveness of the collaborative approach that we followed. It must also be stressed that virtually all the records presented here are recent. Only five of them date from before 2013 and the bulk of the remaining ones date from 2020-2023. Thus, they yield information on the present, as opposed to historical, bee fauna of Italy.

Various recent additions to the bee fauna of Italy come from western Liguria or south-western Piemonte and concern species with mostly west Mediterranean or west European distribution, such as *Andrenaasperrima* Pérez, 1895 (Carisio et al. 2018), *Andrenarhenana* Stoeckhert, 1930 (Gamba and Carta 2020) and *Dasypodacrassicornis* Friese, 1896 (Bonifacino 2021). This pattern is repeated in the present study with the discovery of *Megachileopacifrons* in western Liguria and of *Anthidiellumbreviusculum* in the Val di Susa in Piemonte. Many other western species show a roughly similar distribution, occurring in France almost up to the Italian border. Several of them are likely to occur also in Italian territory. One could ask whether a similar situation might hold at the eastern end of northern Italy with species from south-eastern Europe. Unlike Liguria which has been surprisingly neglected, the area around Trieste has been intensively studied in the past by Austrian and German entomologists. Still, faunistic surprises might well occur here too.

Several records resulted from areas previously neglected by bee experts. Two of the species newly reported here, *Coelioxysalatus* and *Megachilelapponica*, were recorded in the Alpine portion of Veneto. This is an area that has been little explored in the past, as is true, surprisingly, for most of the Italian Alps. Here few areas, mostly Trentino-Alto Adige/Südtirol and Valle d’Aosta, have seen significant melittological activity, especially by German, Austrian and Swiss researchers (Hellrigl 2006 and references therein, Kopf 2008, Steinmann 2002). Similarly, several of the species reported in the present study were caught in riverine or humid inland areas. This seems to indicate that these areas have been partly ignored in the past and deserve particular attention.

Finally, peninsular and insular Italy are probably the most interesting parts of the country from a melittological point of view. Here, large areas are almost unexplored: Basilicata, much of Calabria, southern Campania, much of Molise and Puglia, parts of Abruzzo and much of Sardegna. Various records discussed in the present paper come from these areas and one may add to them the recent discovery of *Eucerabreviceps* Friese in Abruzzo (Aubert et al. in press), of *Andrenafreygessneri* Alfken and *A.probata* Warncke in Abruzzo and of *A.oralis* Morawitz in Puglia (Wood et al. 2023). As indicated by our finds of *Hylaeusglacialis*, *H.nivaliformis*, *Lasioglossumsubfulvicorne*, *Megachilelapponica* and other species, the Apennines, particularly in their montane and alpine stages, probably harbour a faunistic richness that we have only begun to explore.

We have hinted at the effectiveness of the collaborative methods adopted by our network. Reliable identifications were achieved via accurate morphological evaluation with exchange of photographic documentation and specimens and with the help of up-to-date literature, discussion with the involvement of specialists and DNA barcoding when possible. As in other similar contexts (Cornalba et al. 2020, Biella et al. 2022), DNA-based identification techniques proved to be a very useful means of confirming the identity of challenging specimens, taking advantage of a well populated European reference dataset in BOLD. In turn, our study contributed to the BOLD reference dataset by producing regionally important sequences (e.g. first Italian reference barcodes) or first reference sequences for species that had never been barcoded before.

As we mentioned in the Introduction, precise data on the bee fauna of Italy are scattered and often of difficult access. It would be highly desirable to collect and organise as many as possible of the available data in a publicly accessible database, patterned for instance after the Swiss National Apoidea Databank (Praz et al. 2022), other possible examples being the Database of Iberian bees (Bartomeus et al. 2022) or the Wild Bees of Chile project (López‐Aliste et al. 2021). This would be important not only for research purposes, but also because it would provide an essential tool for the evaluation of conservation needs and for the planning of conservation actions. A central node should be created that would coordinate the databasing of public and private collections. The node should be provided by a public or private institution that can guarantee a robust IT infrastructure and a continuity of effort over the years. Arrangements should be made for continuing the process of digitising entomological collections to populate the database. Experience, including the present paper, indicates that much valuable material is held in private and research collection. The owners of these collections should be encouraged to confer their data to the central node. The most difficult part will probably be the quality control of the data in order to validate the identifications and the metadata, as experience indicates that misidentifications are particularly frequent in various Italian collections. Correcting identification errors would require a major effort, for which Italy is ill-equipped, given the scarcity of taxonomic expertise available in the country. However, with this study, we hope to stimulate the interest of taxonomists, collection owners and curators, administrators and conservationists in building a reliable source of data of bee occurrence in Italy in the near future.

## Supplementary Material

XML Treatment for Colletes acutus

XML Treatment for Hylaeus glacialis

XML Treatment for Hylaeus nigrifacies

XML Treatment for Hylaeus nivaliformis

XML Treatment for Andrena alutacea

XML Treatment for Andrena amieti

XML Treatment for Andrena ampla

XML Treatment for Andrena binominata

XML Treatment for Andrena bucephala

XML Treatment for Andrena compta

XML Treatment for Andrena confinis

XML Treatment for Andrena nigroviridula

XML Treatment for Andrena semilaevis

XML Treatment for Halictus carinthiacus

XML Treatment for Lasioglossum algericolellum

XML Treatment for Lasioglossum monstrificum

XML Treatment for Lasioglossum strictifrons

XML Treatment for Lasioglossum subaenescens

XML Treatment for Lasioglossum subfulvicorne

XML Treatment for Seladonia gavarnica

XML Treatment for Macropis europaea

XML Treatment for Anthidiellum breviusculum

XML Treatment for Chelostoma grande

XML Treatment for Coelioxys alatus

XML Treatment for Megachile lapponica

XML Treatment for Megachile opacifrons

XML Treatment for Megachile semicircularis

XML Treatment for Osmia heteracantha

XML Treatment for Pseudoanthidium stigmaticorne

XML Treatment for Rhodanthidium siculum

XML Treatment for Rhodanthidium sticticum

XML Treatment for Trachusa integra

XML Treatment for Ammobates vinctus

XML Treatment for Anthophora affinis

XML Treatment for Anthophora calcarata

XML Treatment for Anthophora dufourii

XML Treatment for Anthophora femorata

XML Treatment for Anthophora fulvitarsis

XML Treatment for Bombus hypnorum

XML Treatment for Epeoloides coecutiens

XML Treatment for Epeolus productulus

XML Treatment for Eucera furfurea

XML Treatment for Eucera pannonica

XML Treatment for Eucera terminata

XML Treatment for Nomada duplex

XML Treatment for Nomada flavopicta

XML Treatment for Nomada hungarica

XML Treatment for Tetralonia inulae

CB89EBF8-F8F9-5F58-83D3-13CFCDEA6749 10.3897/BDJ.12.e116014.suppl1 Supplementary material 1 Table of all specimens subjected to DNA barcoding Data type Occurrences and DNA specifics File: oo_939867.xlsx https://binary.pensoft.net/file/939867 Maurizio Cornalba, Marino Quaranta, Marco Selis, Simone Flaminio, Sirio Gamba, Maurizio Mei, Marco Bonifacino, Andree Cappellari, Roberto Catania, Pietro Niolu, Stefano Tempesti, Paolo Biella

FB29C219-1086-531B-B64F-E6C66DA0CD34 10.3897/BDJ.12.e116014.suppl2 Supplementary material 2 Additional pictures of diagnostic features of selected species Data type Images File: oo_937478.pdf https://binary.pensoft.net/file/937478 Maurizio Cornalba, Marino Quaranta, Marco Selis, Simone Flaminio, Sirio Gamba, Maurizio Mei, Marco Bonifacino, Andree Cappellari, Roberto Catania, Pietro Niolu, Stefano Tempesti, Paolo Biella

## Figures and Tables

**Table 1.** List of recorded species and level of faunistic novelty (abbreviations: IT= Italy, MI = mainland Italy, Ab = Abruzzo, Ca = Calabria, Cp = Campania, ER = Emilia-Romagna, La = Lazio, Li = Liguria, Lo = Lombardia, Pi = Piemonte, Pu = Puglia, To = Toscana, Sa = Sardegna, Si = Sicilia, Um = Umbria, Ve = Veneto); species in bold are new for Italy.

**Family**	**Species**	**New for**
Colletidae	*Colletesacutus* Pérez, 1903	MI, Pu
	*Hylaeusglacialis* Morawitz, 1872	La
	*Hylaeusnigrifacies* Bramson, 1879	ER, La
	*Hylaeusnivaliformis* Dathe, 1977	La
Andrenidae	*Andrenaalutacea* Stoeckhert, 1942	Lo
	*Andrenaamieti* Praz, Müller, Genoud, 2019	La
	*Andrenaampla* Warncke, 1967	Li
	*Andrenabinominata* Smith, 1853	MI, Pu
	*Andrenabucephala* Stephens, 1846	La
	*Andrenacompta* Lepeletier, 1841	MI, Ca
	***Andrenaconfinis* Stoeckhert, 1930**	IT, MI, Lo
	*Andrenanigroviridula* Dours, 1873	Ca, Um
	*Andrenasemilaevis* Pérez, 1903	La
Halictidae	*Halictuscarinthiacus* Blüthgen, 1936	Ve
	***Lasioglossumalgericolellum* (Strand, 1909)**	IT, Si
	*Lasioglossummonstrificum* (Morawitz, 1891)	ER, La
	*Lasioglossumstrictifrons* (Vachal, 1895)	MI, La
	*Lasioglossumsubaenescens* (Pérez, 1896)	La
	*Lasioglossumsubfulvicorne* (Blüthgen, 1934)	ER
	*Seladoniagavarnica* (Pérez, 1903)	La
Melittidae	*Macropiseuropaea* Warncke, 1973	La
Megachilidae	***Anthidiellumbreviusculum* (Pérez, 1890)**	IT, MI, Pi
	*Chelostomagrande* (Nylander, 1852)	Li
	***Coelioxysalatus* Foerster, 1853**	IT, MI, Ve
	***Megachilelapponica* Thomson, 1872**	IT, MI, Ab, Ve
	***Megachileopacifrons* Pérez, 1897**	IT, MI, Li
	***Megachilesemicircularis* auct. nec Zanden, 1996**	IT, MI, La
	*Osmiaheteracantha* Pérez, 1896	Sa
	*Pseudoanthidiumstigmaticorne* (Dours, 1873)	La, Li
	*Rhodanthidiumsiculum* (Spinola, 1838)	MI, Ca
	*Rhodanthidiumsticticum* (Fabricius, 1787)	MI, Ca, Li
	***Trachusaintegra* (Eversmann, 1852)**	IT, MI, La, Sa
Apidae	*Ammobatesvinctus* Gerstaecker, 1869	La
	*Anthophoraaffinis* Brullé, 1832	Li
	*Anthophoracalcarata* Lepeletier, 1841	
	*Anthophoradufourii* Lepeletier, 1841	
	*Anthophorafemorata* (Olivier, 1789)	La
	*Anthophorafulvitarsis* Brullé, 1832	Li
	*Bombushypnorum* (Linnaeus, 1758)	Ab
	*Epeoloidescoecutiens* (Fabricius, 1775)	La
	*Epeolusproductulus* Bischoff, 1930	La
	*Eucerafurfurea* Vachal, 1907	Pu
	*Eucerapannonica* Mocsáry, 1878	Ab, La
	*Euceraterminata* Pérez, 1895	La
	*Nomadaduplex* Smith, 1854	ER, La, To
	*Nomadaflavopicta* (Kirby, 1802)	Si
	*Nomadahungarica* Dalla Torre & Friese, 1894	Ab, La, Li
	*Tetraloniainulae* Tkalců, 1979	ER, Lo
