# Supplementary material for: Exploring the hidden riches: Recent remarkable faunistic records and range extensions in the bee fauna of Italy (Hymenoptera, Apoidea, Anthophila)
Source: Biodivers Data J. 2024 Feb 16;12:e116014. doi: 10.3897/BDJ.12.e116014 (PMC10892157; doi:10.3897/BDJ.12.e116014)
Supplement: Supplementary material 2 — Additional pictures of diagnostic features of selected species [file bdj-12-e116014-s002.pdf]

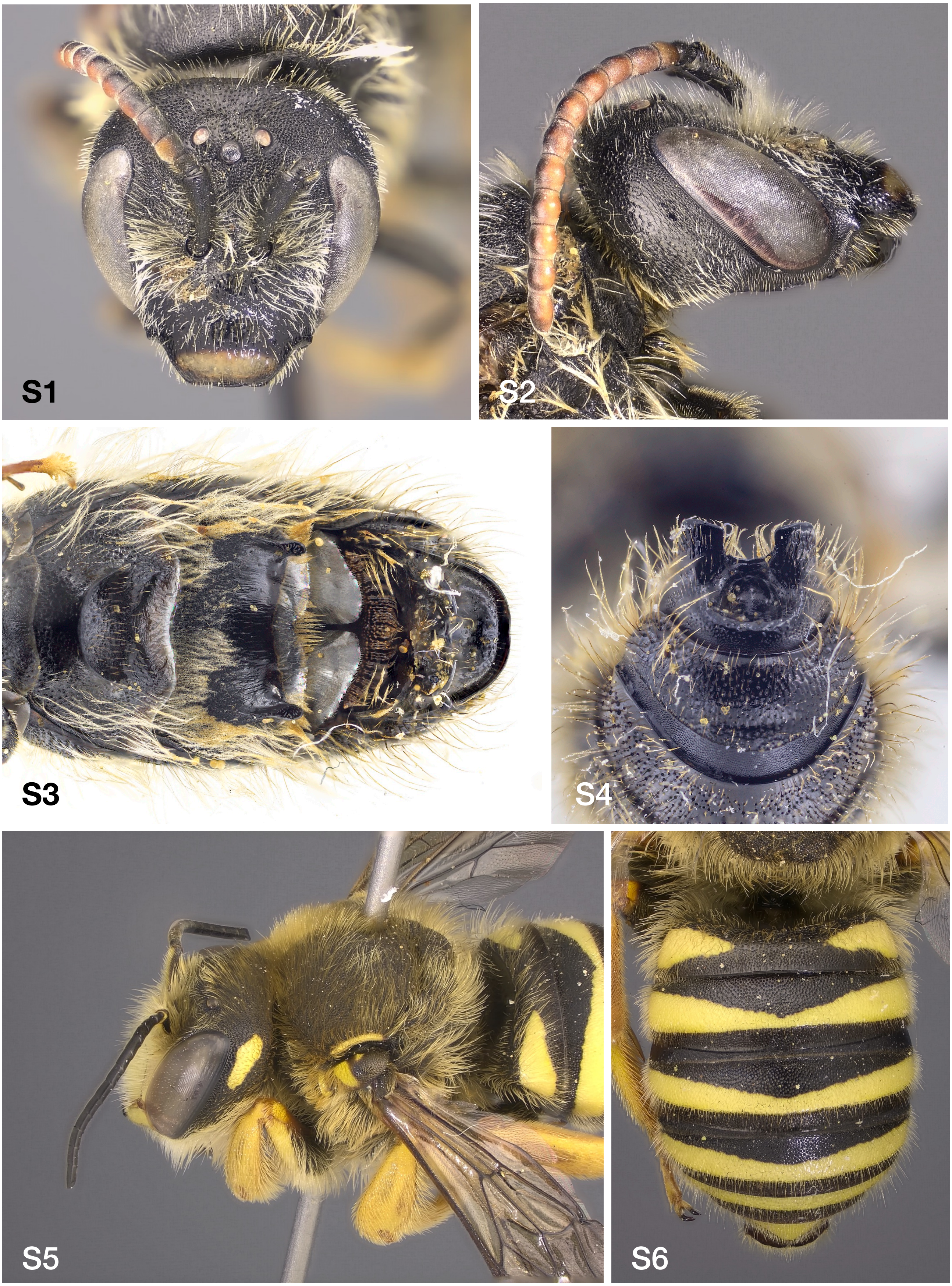

Figures S1–S6. S1, S2 *Halictus carinthiacus* Blüthgen, 1936 male from Belluno **S1** head frontal **S2** head lateral **S3, S4** *Chelostoma grande* (Nylander, 1852) male from Rocchetta Nervina **S3** sterna **S4** last tergites **S5, S6** *Trachusa integra* (Eversmann, 1852) male from Roma **S5** mesosoma **S6** tergites

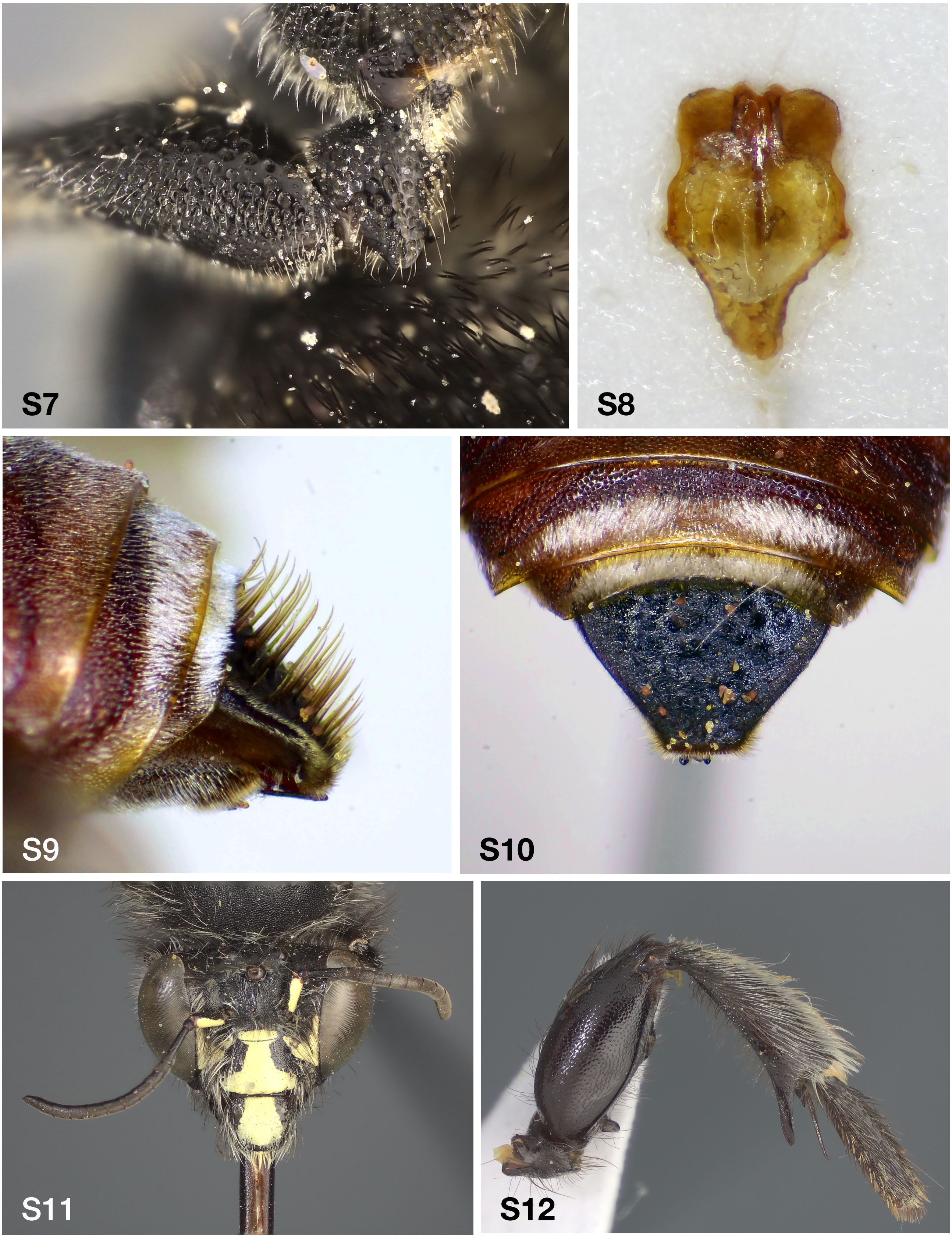

Figures S7–S12. S7 *Osmia heteracantha* Pérez, 1896 female from Oristano, hind trachanter **S8** *Ammobates vinctus* Gerstaecker, 1869 male from Norchia, sternite 8 **S9, S10** *Ammobates vinctus* Gerstaecker, 1869 female from Norchia, last tergites **S9** lateral **S10** dorsal **S11, S12** *Anthophora calcarata* Lepeletier, 1841 male from Pantelleria **S11** face **S12** hind leg

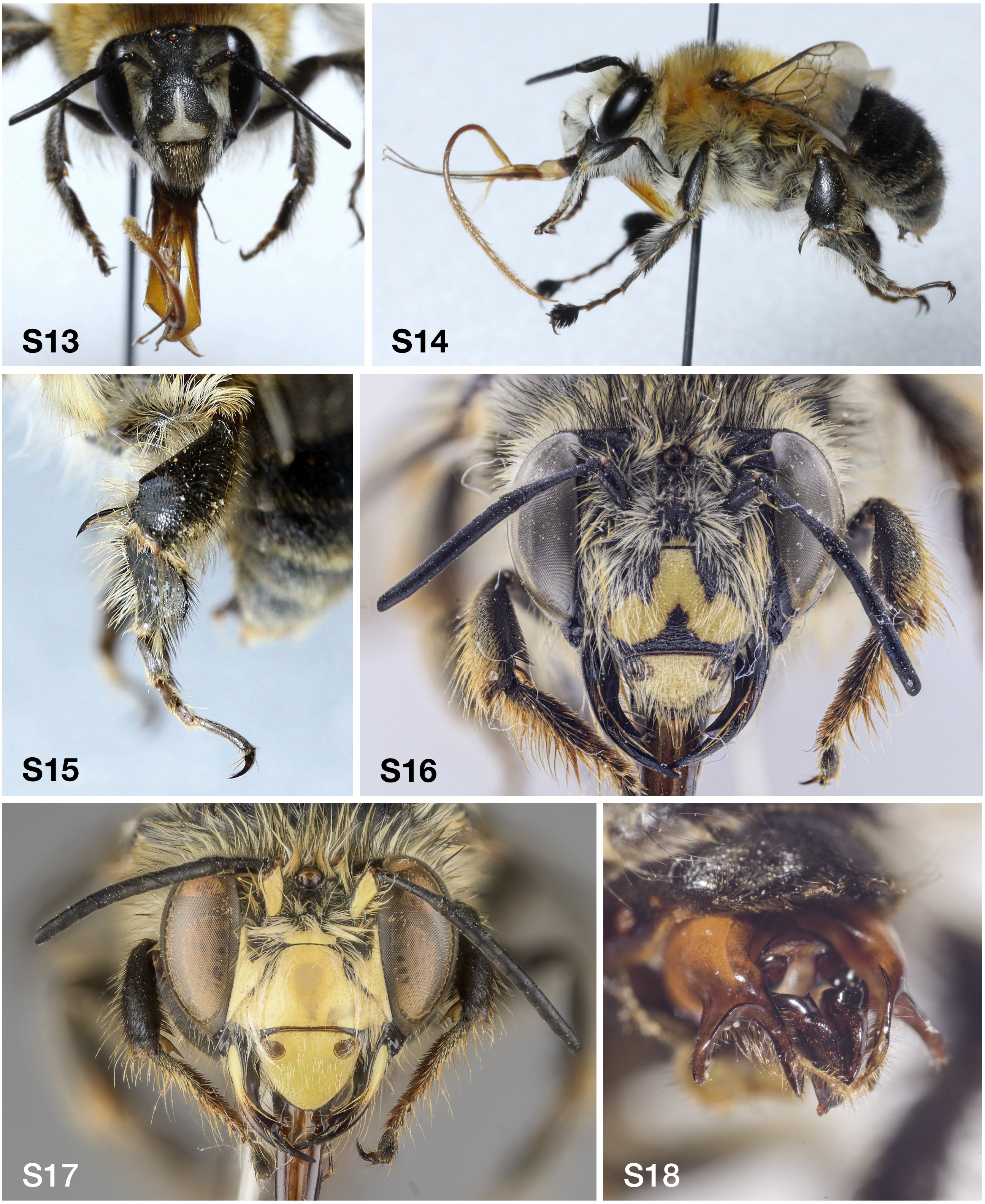

Figures S13–S18. S13 *Anthophora dufourii* Lepeletier, 1841 female from Dego, frontal **S14, S15** *Anthophora dufourii* Lepeletier, 1841 male from Dego **S14** habitus lateral **S15** hind tibia and tarsi **S16** *Anthophora fulvitarsis* Brullé, 1832 female from Ventimiglia, head **S17, S18** *Anthophora fulvitarsis* Brullé, 1832 male from Ventimiglia **S17** head **S18** genitalia

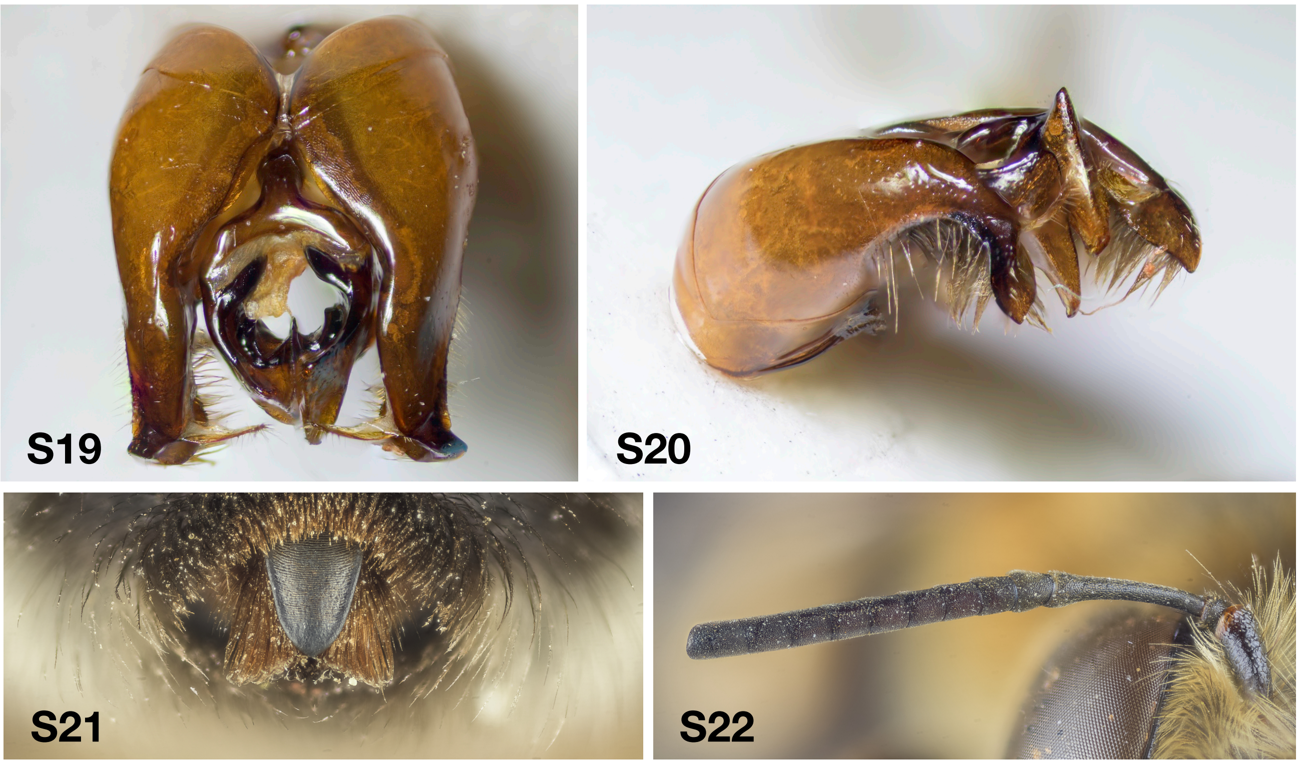

Figures S19–S22. *Anthophora affinis* Lepeletier, 1841 **S19, S20** male from Apricale, genitalia **S19** dorsal **S20** posterolateral **S21, S22** female from Camporosso **S21** pygidial plate **S22** antenna
